# Microgel Encapsulated Mesoporous Silica Nanoparticles for Releasing Wnt16 to Synergistically Treat Temporomandibular Joint Osteoarthritis

**DOI:** 10.1002/advs.202404396

**Published:** 2024-09-09

**Authors:** Yan Zhu, Lingyan Cao, Mu Yuan, Xuzhuo Chen, Xinru Xie, Minhan Li, Chi Yang, Xiansong Wang, Zhigui Ma

**Affiliations:** ^1^ Department of Oral Surgery Shanghai Ninth People's Hospital College of Stomatology Shanghai Jiao tong University School of medicine National Clinical Research Center for Oral Diseases Shanghai Key Laboratory of Stomatology Shanghai Research Institute of Stomatology Research Unit of Oral and Maxillofacial Regenerative Medicine, Chinese Academy of Medical Sciences Shanghai 200011 China; ^2^ National Center for Stomatology National Clinical Research Center for Oral Diseases Shanghai Key Laboratory of Stomatology Shanghai Engineering Research Center of Advanced Dental Technology and Materials Shanghai 200011 China; ^3^ Department of Plastic and Reconstructive Surgery Shanghai Key Laboratory of Tissue Engineering Shanghai Ninth People’s Hospital, Shanghai Jiao Tong University School of Medicine Shanghai 200011 China

**Keywords:** biofunctional hydrogel, mesoporous silica nanoparticle, temporomandibular joint osteoarthrosis, Wnt16, Wnt/β‐catenin

## Abstract

Temporomandibular joint osteoarthritis (TMJOA) is a commonly encountered degenerative joint disease in oral and maxillofacial surgery. Recent studies have shown that the excessive unbalanced activation of Wnt/β‐catenin signaling is connected with the pathogenesis of TMJOA and due to the inability to inhibit the over‐activated Wnt pathway, while Wnt16‐deficient mice has a more severe Knee OA. However, the efficacy of direct intra‐TMJ injection of Wnt16 for the relief of TMJOA is still not directly confirmed. Moreover, small‐molecule drugs such as Wnt16 usually exhibit short‐lived efficacy and poor treatment adherence. Therefore, in order to obtain a stable release of Wnt16 both in the short and long term, this study fabricates a double‐layer slow‐release Wnt16 carrier based on mesoporous silica nanospheres (MSNs) encased within hyaluronic acid (HA) hydrogels. The biofunctional hydrogel HA/Wnt16@MSN is analyzed both in vitro and in vivo to evaluate the treatment of TMJOA. As a result, it shows superior pro‐cartilage matrix restoration and inhibition of osteoclastogenesis ability, and effectively inhibits the over‐activation of the Wnt/β‐catenin pathway. Taken together, biofunctional hydrogel HA/Wnt16@MSN is a promising candidate for the treatment of TMJOA.

## Graphical abstract

A double layer slow‐release Wnt16 carrier based on mesoporous silica nanospheres (MSNs) encased within hyaluronic acid (HA) hydrogels is designed for treating temporomandibular joint osteoarthritis (TMJOA). This biofunctional hydrogel HA/Wnt16@MSN shows superior pro‐cartilage matrix restoration and inhibition of osteoclastogenesis ability, and effectively inhibited the over‐activation of the Wnt/β‐catenin pathway.

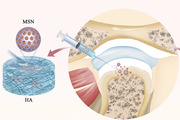

## 1 Introduction

TMJOA is a joint degenerative disease that is commonly encountered in oral and maxillofacial medicine and is a leading cause of chronic disability.^[^ ^1^, ^2^ ^]^ At present, the pathogenesis of TMJOA remains poorly understood. Multifactorial processes including inflammation, aging, chondrocyte senescence, genetic factors, and joint overload are considered to be potential triggers of TMJOA.^[^ ^3^, ^4^, ^5^, ^6^ ^]^ These factors lead to the development of typical pathologic features such as cartilage degradation, subchondral sclerosis, and chronic synovitis.^[^ ^7^ ^]^ The current goals and principles regarding the treatment of TMJOA are to reduce patients' symptoms, such as swelling and joint pain and, and to improve joint function. However, existing treatments, both conservative and surgical, are unsatisfactory and irreversible. For example, non‐steroidal anti‐inflammatory drugs (NSAIDs), disease‐modifying anti‐rheumatic drugs (DMARDs), and other small‐molecule drugs are mostly used for short‐term treatment of the symptoms,^[^ ^8^, ^9^, ^10^ ^]^ but their adverse side effects due to frequent administration and high dosages, can be as harmful as the condition. As a result, there is an urgent demand for new effective, and minimally invasive TMJOA therapies that restore joint function and prevent its progression.

Recent studies have shown that the expression of Wnt16 is significantly upregulated in post‐traumatic cartilage, and transgenic mice lacking Wnt16 expression present severe OA.^[^ ^11^ ^]^ It is reported that Wnt16 plays a crucial role in preserving the equilibrium of canonical Wnt/β‐catenin signaling, thereby averting detrimental and excessive activation. This function assists in maintaining the homeostasis of progenitor cells. In addition, Wnt16 has been shown to suppress the RANKL‐induced degeneration of OCs through Wnt/β‐catenin signaling.^[^ ^12^ ^]^ The activation and conduction of a series of signaling pathways are connected to the development of OA pathology and cartilage degeneration. Recent studies have revealed that the Wnt/β‐catenin pathway is significant in the regulation of chondrocyte proliferation, ossification, and degeneration.^[^ ^13^ ^]^ For instance, Nalesso et al.^[^ ^11^ ^]^ reported that Wnt16 plays a balancing role in the classical Wnt pathway, preventing its overactivation to maintain chondrogenic stem cell homeostasis. And it has also been shown that rhWnt16 has anti‐inflammatory and protective effects on chondrocytes and cartilage explants in recent studies.^[^ ^14^, ^15^ ^]^ However, all of the above studies were conducted to investigate the role of Wnt16 in knee osteoarthritis. And there are no studies that directly confirmed the alleviating effect of Wnt16 on TMJOA. Not only that, but the molecular weight of Wnt16 is only 37 kDa, which makes it unavoidably reactive with components of the joint fluid. Thus, it is easily degraded and cleared from the TMJ cavity if administered by conventional in situ injection. Therefore, drug‐delivery systems are always needed to assist in drug release. In general, an ideal drug delivery system should be good at allowing controlled drug release with excellent biocompatibility and benefit drug potency. To better control the release of Wnt16, we used MSN particles to encapsulate Wnt16 to obtain a long duration of action in its joint cavity. With the development of nanotechnology, MSNs have been shown to exhibit unique properties, such as high specific surface area, large pore volume, adjustable pore‐size range, and easy surface modification with organic groups, all of which can be exploited for controlled drug release of biologically active substances.^[^ ^16^, ^17^, ^18^ ^]^ For example, a hyaluronan synthase combined with a novel mesoporous silica material loading system (MSN‐HAS2) was constructed, and a series of in vivo and in vitro experiments showed that MSNs can improve the synthesis and secretion of high‐molecular‐weight HA and reduce inflammation by transporting hyaluronan synthase into the synovial cells of the TMJ, which is conducive to the treatment of TMJOA.^[^ ^19^ ^]^

Meanwhile, in order to better accomplish intra‐articular injection and play the role of flushing the joint cavity to alleviate inflammation, this study used HA as a carrier to form a double‐layer wrapping structure of HA/Wnt16@MSN. In recent years, HA has become recognized as an essential bio‐functional material involved in cartilage matrix repair and that is a key regulator of chondrocyte differentiation. It is widely distributed in human joints and connective tissues, it is naturally derived from non‐sulfated glycosaminoglycan, and it is a nonfibrillar and nonimmunogenic material. HA occurs physiologically within the synovial fluid, helping to maintain mechanical strength, fibrocartilage, and chondrocyte function.^[^ ^20^ ^]^ Due to its superior biocompatibility, functional HA hydrogels are widely used in clinical TMJOA treatment. However, its rapid clearance from affected joints remains a critical limitation to its drug efficacy.^[^ ^21^, ^22^ ^]^

**Figure 1:**
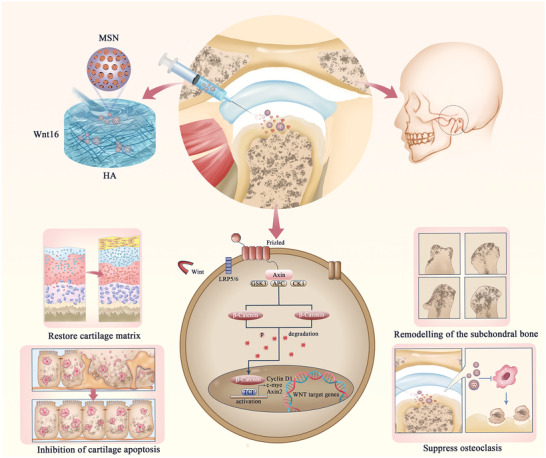
Schematic illustration of application and mechanism of the HA/Wnt16@MSN system for synergistic therapy in TMJOA by regulating Wnt/βcatenin signaling.

Because of the similarities between the inflammatory microenvironment of knee osteoarthritis (KOA) and TMJOA, it is reasonable to introduce Wnt16, which has been shown to be critically important in KOA, in the treatment of TMJOA. Accordingly, in the present study, the “two‐stage” drug delivery system HA/Wnt16@MSN was constructed. It consists of three key components; a hydrogel matrix, MSNs, and Wnt16. The hydrogel serves as a critical component for proteoglycans of the extra‐cellular matrix (ECM) and allows highly localized drug release (first‐stage release); the MSNs act as carriers to provide long‐lasting relief in joints by the slow release of Wnt16 (second‐stage release), as well as resolving early inflammation in chondrocytes; and Wnt16 promotes cartilage matrix synthesis and has an anti‐inflammatory effect by activating Wnt/β‐catenin signaling. Research conducted in vivo using a rat model of TMJOA amply established the efficacy of HA/Wnt16@MSN in protecting articular cartilage via a combination of anti‐inflammatory effects and cartilage matrix restoration The synthesis schematic is shown in **Figure** **1**. In summary, HA/Wnt16@MSN, as a novel multifunctional composite drug delivery system that can be used with an IA injection approach to efficiently alleviate TMJOA.

## 2 Results and Discussion

### 2.1 Preparation and Characterization of HA/Wnt16@MSN

**Figure 2:**
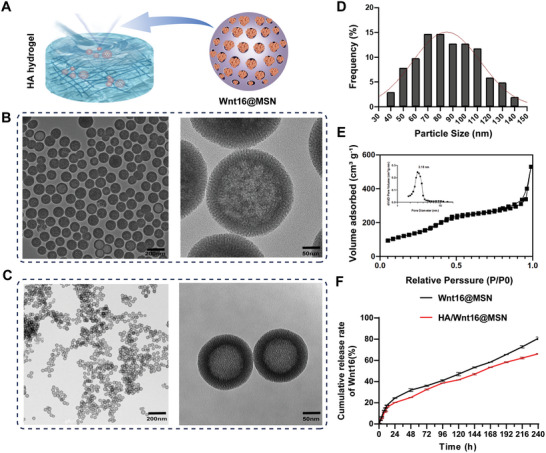
Characterization of MSNs and Wnt16@MSN. A) Schematic representation of the HA/Wnt16@MSN synthesis process. B) Representative images of SEM and TEM images of MSNs. C) Representative images of SEM and TEM images of Wnt16 encapsulated in MSNs. D) The pore size distributions of bare MSNs and N_2_ adsorption–desorption isotherms. E) N_2_ adsorption and desorption isotherms for MSNs. F) Cumulative release curve of Wnt16 encapsulated in MSNs and Wnt16 encapsulated in MSN loaded by HA.

The schematic of the constructed composite nanoparticle‐functionalized hydrogel is shown in **Figure** **2**A. To obtain high‐purity MSNs, we used aerosol preparation as stated in the literature.^[^ ^19^ ^]^ Scanning electron microscopy (SEM) photos of the MSNs show pores on the surface of the particles, while their transmission electron microscopy (TEM) images (Figure 2B) show that the particles have a bilayer structure with a mesoporous layer of 11.99 ± 1.34 nm thickness on the surface. The average particle size is 86.53 ± 23.74 nm (Figure 2D). All these results demonstrate the successful synthesis of Wnt16‐loaded MSNs with regular morphology, good dispersion, and uniform particle size.

As is common for mesoporous silica, the N_2_ adsorption–desorption isotherms of MSNs are type IV (Figure 2E), indicating the presence of mesopores with regular shapes and highly homogeneous pore sizes. The pore size of the nanomaterials obtained by the Barrett–Joyner–Halenda (BJH) method was 6.27 nm, the pore volume was 0.851583 cm^3^ g^−1^, and the Brunauer–Emmett–Teller (BET)‐calculated specific surface area was 477.3775 m^2^ g^−1^.

Wnt16 was loaded into the MSNs as described in the Methods section. Representative SEM and TEM images of MSNs loaded with Wnt16 are shown in Figure 2C. The micro bicinchoninic acid (BCA) method was used to measure the Wnt16 loading, adsorption, and release rates of Wnt16@MSN, revealing that the system provides controlled Wnt16 delivery, with 80% release over 10 days in vitro. In addition to this, we also measured the cumulative release rate of Wnt16 by HA/Wnt16@MSN, which was 65% after 10 days. Meanwhile, the results of slow release of Wnt16 by HA alone showed that Wnt16 was close to being completely released after 48 h (Figure S7, Supporting Information).

These results show that this bio‐nano hydrogel can achieve sustained drug release, which is important for achieving a persistent therapeutic effect (Figure 2F).

MSNs possess a hollow and porous structure, enabling the encapsulation of a significant quantity of bioactive proteins.^[^ ^23^ ^]^ Recently, several studies have shown that MSNs can facilitate the controlled release of various drugs, including ibuprofen, erythromycin, vancomycin, and alendronate.^[^ ^24^, ^25^, ^26^ ^]^ Thus, MSN‐encapsulated‐Wnt16 nanoparticles could increase the shelf‐life of the Wnt16 preparation between formulation and administration and could prolong the release of Wnt16, thus prolonging its biological activity. And the drug‐loading efficiency (LE) and encapsulation efficiency (EE) were calculated by the following equations:

(1) LE%×100%=1.14%

(2) EE%=×100%=73.64%

### 2.2 HA/Wnt16@MSN Biocompatibility and Effects On Chondrocytes In Vitro

**Figure 3:**
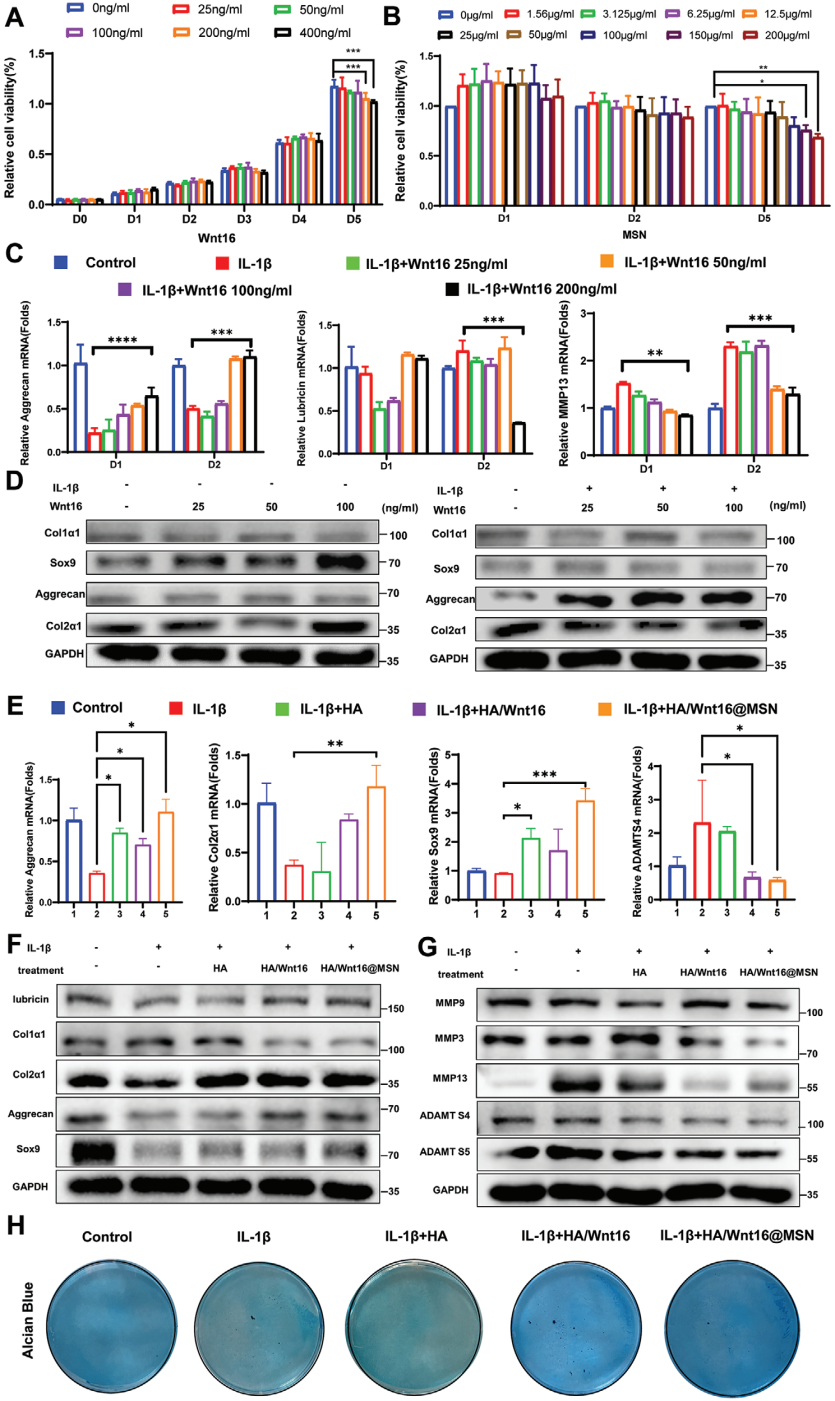
Evaluation of the biocompatibility HA/Wnt16@MSN in vitro and its inflammation suppression effects. A) CCK‐8 analysis of chondrocytes cocultured with various concentrations of Wnt16 at different time durations. B) CCK‐8 analysis of chondrocytes cocultured with various concentrations of MSNs at different time durations. C) qRT‐PCR analysis for the mRNA expression levels of aggrecan, lubricin, and MMP13 in chondrocytes with concentrations of Wnt16 at different time durations. D) Western blot analysis for the protein expression levels of cartilage repair‐related protein in chondrocytes with concentrations of Wnt16 at different time durations. E) qRT‐PCR analysis for the mRNA expression levels of aggrecan, col2, Sxo9, and ADAMTS4 in chondrocytes in various treatment groups. F) Western blotting analysis for the protein expression levels of cartilage repair‐related protein in chondrocytes in various treatment groups. G) Western blotting analysis for the protein expression levels of cartilage inflammation‐related protein in chondrocytes in various treatment groups. H) Alcian Blue staining in chondrocytes with various treatment groups. (*n* = 3 independent samples, *p < 0.05; **p < 0.01; ***p < 0.001; ****p < 0.0001).

To obtain the smooth release of Wnt16, the double‐layer wrapping structure was prepared as above. Wnt16 is a protein expressed predominantly in the superficial cartilage of the condyle in rat temporomandibular joints (Figure S1, Supporting Information). Interestingly, we found that the level of Wnt16 protein increases in the short term (less than 24 h) after the cytokine IL‐1β was applied to the SW1353 cells to induce inflammation, which appears to induce a temporary state of cellular stress (Figures S2 and S3, Supporting Information). In contrast, Wnt16 protein levels decrease in response to prolonged inflammatory stimulation (longer than 24 h up to 72 h; Figure S4, Supporting Information). We confirmed this in the long‐term rat TMJOA model (Figure S5, Supporting Information). SW1353 chondrocytes treated with different concentrations of Wnt16 (0–400 ng mL^−1^; **Figure** **3**A) and MSNs (0–200 µg mL^−1^ ; Figure 3B) were subjected to CCK‐8 assay and live/dead staining (Figure S6, Supporting Information) to determine cell viability. The CCK‐8 results (Figure 3B) show that the viability of the chondrocytes is decreased at an MSN concentration of 150 µg mL^−1^ without inflammatory stimulation, Wnt16 inhibits the proliferation of chondrocytes at a concentration of 200 ng mL^−1^, indicating the optimum concentration of Wnt16 is 100 ng mL^−1^.

The results of live/dead staining show that the number of chondrocytes decreases with increasing MSN concentration, and the morphology of the yellow‐green labeled live cells does not change significantly, even after treatment at the high concentration of 200 µg mL^−1^ for 48 h. However, after 5 days of incubation at a concentration of 200 µg mL^−1^, the chondrocytes show obvious wrinkling and deformation, and the number of red‐stained dead cells is significantly increased.

### 2.3 Wnt16 and MSNs Promote Cartilage Matrix Synthesis and Inhibit Its Degradation In Vitro

Chondrocytes were cocultured with different concentrations of Wnt16 and MSNs, and the expression of genes and proteins related to cartilage synthesis and degradation in chondrocytes were explored by quantitative real‐time polymerase chain reaction (qRT‐PCR) and Western blot (WB) experiments to determine the optimal Wnt16 and MSN concentrations and their ratio. The qRT‐PCR results (Figure 3C) show that in chondrocytes under inflammatory stimulation for 24 h, Wnt16 upregulates the expression of genes against chondrogenic matrix aggrecan and lubricin. Conversely, in the absence of inflammation stimulation, MSNs at concentrations below 200 µg mL^−1^ are not pro‐inflammatory. However, under IL‐1β‐induced inflammation conditions, the gene expression of inflammation‐related factor MMP13 is upregulated, and the increased expression of MMP13 is decreased with increasing MSN concentration (Figure S8 and S9, Supporting Information).

Wnt16 at 100 ng mL^−1^ significantly promotes the protein expression of Sox9 and Col2 in chondrocytes under non‐inflammatory conditions as compared with the other treatment groups. In chondrocytes incubated with IL‐1β, Wnt16 significantly promotes aggrecan protein expression and decreases Sox9 expression (Figure 3D).

Silicon is an essential trace element for the development of healthy human bone tissue, cartilage tissue, and connective tissue. It is also involved in regulating the physiological process of osteogenesis and has a significant impact on bone metabolism. Accordingly, silicon deficiency leads to bone defects and deformations. Silicon in the human body mainly comes from the diet. However, in addition to dietary sources, a variety of silicon‐containing bioactive materials have been employed in the field of bone tissue regeneration and repair,^[^ ^27^ ^]^ which have been shown to promote osteogenesis and angiogenesis. Mechanistically, it is thought that silicone‐containing bioactive materials activate signaling pathways related to stem cell osteogenesis by releasing silicone‐containing ionic products, which have been shown to have a beneficial effect on osteogenesis and angiogenesis in vivo.^[^ ^28^ ^]^

However, the effects of silicon and its bioactive forms on cartilage remain unclear. Thus, we have examined the pro‐matrix repair and anti‐inflammatory properties of Wnt16@MSN on cartilage in the current work.

### 2.4 HA/Wnt16@MSN Relieves Inflammation of Chondrocytes In Vitro

From the results presented above, we determined the optimal configuration of the composite hydrogel to be a Wnt16 concentration of 100 ng mL^−1^, an MSN concentration of 100 µg mL^−1^, and an HA concentration of 10 mg mL^−1^ (as used most commonly in the clinic). The concentration and loading of the components of the composite are shown schematically in Figure S9 (Supporting Information). In addition, the results of qRT‐PCR experiments (Figure 3E) show that the expression of Sox9 and Col2 significantly decreased in the IL‐1β group after 48 h of culture with chondrocytes. Under inflammatory stimulation, the expression of Sox9 in the Wnt16 and Wnt16/MSN groups is noticeably higher than that in the IL‐1β group.

The WB results are consistent with the qRT‐PCR results (Figure 3F,G) showing that at the protein level, HA/Wnt16@MSN likewise had the strongest restorative effect on cartilage matrix synthesis proteins and the strongest inhibitory effect on cartilage inflammation marker proteins. The results of Alcian Blue staining show a decrease in cartilage matrix synthesis after inflammatory stimulation (Figure 3H). However, with the protective effect of HA/Wnt16@MSN, cartilage matrix synthesis is somewhat restored.

In our initial exploration of the role of Wnt16 on chondrocytes, we found that Wnt16 expression is upregulated at both mRNA and protein levels as a result of short‐term inflammatory stimulation, which is consistent with previous reports and confirms that Wnt16 shields chondrocytes against the inflammatory response brought on by IL‐1β.^[^ ^14^ ^]^ Interestingly, we also observed that, after a short period of increased expression following stimulation, the expression of Wnt16 gradually declines. It has been reported that Wnt16‐cKO mice experience more severe OA due to the antagonistic effect of Wnt16 on excessive canonical Wnt signaling activation.^[^ ^11^ ^]^ Accordingly, it is reasonable to speculate that in our study, Wnt16 undergoes long‐term and smooth release after being encapsulated in the double‐layer nanoparticle structure of HA/Wnt16@MSN, effectively preventing the possible chondrocyte stress caused by its sudden release.

### 2.5 HA/Wnt16@MSN Alleviates IL1β‐Induced Chondrocyte Apoptosis

**Figure 4:**
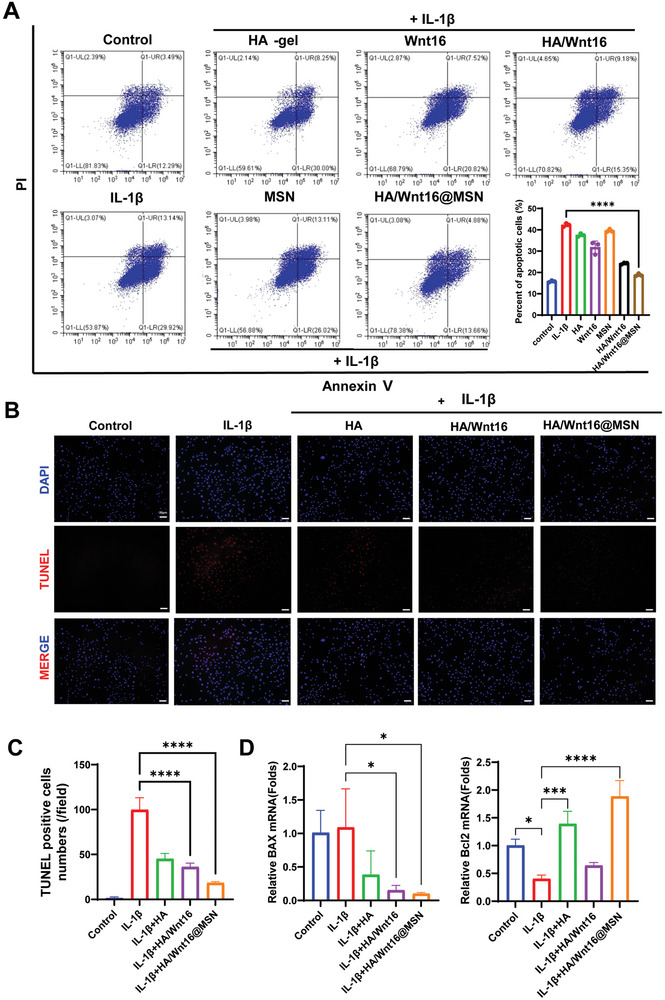
Effect of HA/Wnt16@MSN on inflammatory OCs. A) TRAP staining for OCs with various treatment groups (*n* = 3 independent samples). B) Bone resorption of OCs under different treatment groups by osteo assay plates. C) Number of TRAP‐positive cells. D) Area of TRAP‐positive cells. E) Expression of pro‐inflammatory genes evaluated by RT‐qPCR in OCs. (*p < 0.05; **p < 0.01; ***p < 0.001; ****p < 0.0001).

We evaluated the rescue effect of different components of the composites on IL‐1β‐induced apoptosis in SW1353 cells. The rate of early chondrocyte apoptosis is expressed in the UR region and the rate of late chondrocyte apoptosis is expressed in the LR region, as shown below in **Figure** **4**A. After cocultured with IL‐1β (10 ng mL^−1^) for 24 h, the rate of apoptosis of SW1353 cells is significantly increased compared with the control. When treated with HA/Wnt16@MSN for another 24 h, the apoptosis rate is significantly decreased relative to the IL‐1β‐stimulated group. TUNEL staining indicated that cells in the normal group showed no obvious signs of apoptosis. However, the number of positive cells is significantly higher in the IL‐1β group compared with the negative control group. Treatment with HA/Wnt16@MSN reduces the number of positive cells compared with the IL‐1β group (Figure 4B). The quantitative results of TUNEL staining are presented in Figure 4C. qRT‐PCR analysis showed that IL‐1β stimulation increases the mRNA level of Bax and decreases the mRNA level of BCL2. However, the addition of HA/Wnt16@MSN reverses this trend (Figure 4C).

Therefore, flow cytometry, TUNEL staining, and qRT‐PCR analysis all showed that HA/Wnt16@MSN effectively prevents IL‐1β‐induced apoptosis of SW1353 cells and has potential anti‐apoptotic effects on chondrocytes.

### 2.6 Anti‐Osteoclast Activity of HA/Wnt16@MSN In Vitro

**Figure 5:**
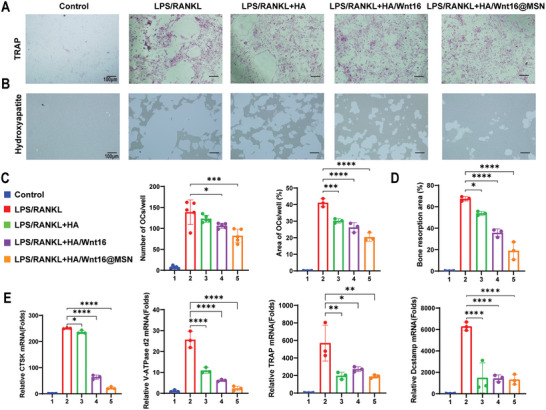
Chondrocytes anti‐apoptotic effect of HA/Wnt16@MSN. A) Annexin V‐FITC staining of different components of HA/Wnt16@MSN in chondrocytes. B) Representative TUNEL staining images and quantitative analysis C) of positive chondrocytes under various treatment groups. D) qRT‐PCR analysis for the mRNA expression levels of Bcl2 and BAX in chondrocytes under various treatment groups. (*n* = 3 independent samples, *p < 0.05; **p < 0.01; ***p < 0.001; ****p < 0.0001).

To further explore the effects of HA/Wnt16@MSN on RANKL‐induced osteoclasts (OCs), hydrogels with various components were added to the cultured cells. In the Tartrate‐resistant acid phosphatase (TRAP) staining assay, a notable increase in TRAP‐positive multinucleated OCs is typically observed after 4 days of stimulation with RANKL. However, the treatment group exhibits a significant reduction in both the number and area of OCs. (**Figure** **5**A). Quantitative analysis of the TRAP staining results is shown in Figure 5C. In addition, in bone assay plates coated with hydroxyapatite, the assessment of bone resorption activity revealed a significant reduction in the area of bone resorption attributed to HA, HA/Wnt16, and HA/Wnt16@MSN treatments (Figure 5B). In particular, when treated with HA/Wnt16@MSN, the bone resorption area is less than 30% that of the single LPS/RANKL stimulation group (Figure 5D).

To further verify the effect of HA/Wnt16@MSN on OCs at the transcriptional level, RT‐qPCR was performed after culturing the Bone marrow‐derived macrophages (BMM) in an OC‐inducing environment for 5 days. Consistently, the expression of OC genes (Trap, V‐ATPase d2, and CTSK, and Dc‐stamp) is significantly upregulated in response to RANKL stimulation, but that for the HA, HA/Wnt16, and HA/Wnt16@MSN groups is significantly reduced (Figure 5E). These results suggest that HA, HA/Wnt16, and HA/Wnt16@MSN exhibit inhibitory effects on OC formation, fusion, and function.

During the process of bone fracture healing, OCs are primarily responsible for the resorption and clearance of damaged bone tissue.^[^ ^29^, ^30^ ^]^ Conversely, in the development of OA, OCs participate in the remodeling and degradation of joint osseous tissue, leading to the loss of articular cartilage.^[^ ^31^, ^32^ ^]^ Wnt16 has been demonstrated to reduce the generation of osteoclasts in vitro through both direct and indirect inhibitory mechanisms.^[^ ^12^, ^33^ ^]^

In this work, we employed MSNs for the first time to deliver Wnt16 in situ. Its sustained and prolonged release inhibits the formation of osteoclasts, contributing to the attainment of a balanced bone metabolism and facilitating bone repair.

### 2.7 HA/Wnt16@MSN Promotes Subchondral Bone Remodeling in TMJOA

**Figure 6:**
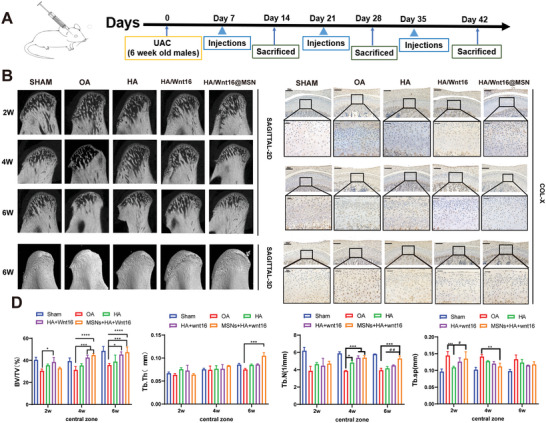
In *vivo* biodistribution and radiological evaluation of HA/Wnt16@MSN on TMJOA. A) Schematic diagram of the in vivo evaluation. B) Representative images of micro‐CT scanning of rat TMJ on 2, 4, and 6w after administration of various treatment groups (*n* = 6). C) immunostaining of COL‐X on 2, 4, and 6w after various treatment groups (*n* = 6). D) Quantitative analysis of micro‐CT scanning of rat TMJ on 2, 4, and 6w after administration of various treatment groups (*n* = 6). (*p < 0.05; **p < 0.01; ***p < 0.001).

To evaluate the therapeutic effects of HA/Wnt16@MSN in vivo, a TMJOA model was established in SD rats induced by unilateral anterior crossbite prosthesis. The morphology of the newly formed bone was analyzed by micro‐computed tomography (micro‐CT) 2, 4, and 6 weeks after injection (**Figure** **6**A). The micro‐CT results (Figure 6B) show that OA changes from sparse to dense trabeculae with osteophyte formation and partial resorption into the deep subchondral bone with time. The HA/Wnt16@MSN group shows a continuous bone cortex, regular trabecular arrangement, and fine structure of subchondral bone trabeculae. Additionally, the HA/Wnt16@MSN group shows BV/TV and Tb values are higher, and Sp is lower, suggesting a discernible increase in subchondral bone volume. On week 6, in comparison to the other three groups, it was found that HA/Wnt16@MSN therapy had the most significant therapeutic effect, demonstrating a substantially full bone surface. (Figure 6D). These data suggest that HA/Wnt16@MSN could attenuate TMJOA in the long term to protect the condylar cartilage and subchondral bone.

Col X is a type of collagen mainly distributed in the hypertrophic layer of the condyle that has been found to act as a substrate for matrix degradation. Col‐X immunostaining (Figure 6C) is strong in the OA group, and its presence alters the properties of the extracellular matrix, possibly acting as a substrate for matrix degradation, endochondral ossification, and vascular invasion, suggesting interference with the synthesis of the cartilage matrix. However, weak staining with a small area is observed for the HA/Wnt16@MSN group. It is mainly distributed in the hypertrophic layer and partially located in the mature layer, suggesting hypertrophy of chondrocytes at a certain stage or in certain areas during OA, without being able to synthesize normal cartilage matrix. The corresponding area for the HA/Wnt16@MSN group is lightly stained with a small area.

### 2.8 HA/Wnt16@MSN Promotes Cartilage Repair in TMJOA

**Figure 7:**
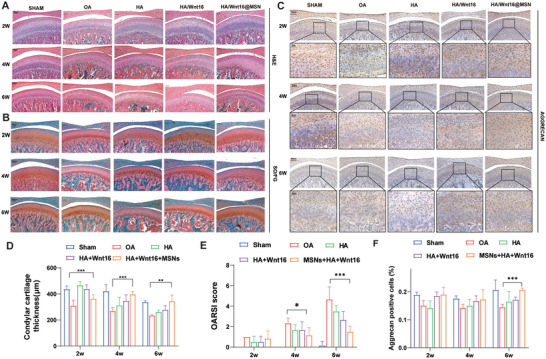
Histological analysis of the therapeutic effect of HA/Wnt16@MSN on TMJOA. A) Representative images of H&E, B)S&F, and C) immunostaining of aggrecan on 2, 4, and 6w after administration of various treatment groups (*n* = 6) D) Quantitative analysis of the condylar cartilage thickness. E) Quantitative analysis of the OARSI score. F) Quantitative analysis of the percentage of aggrecan‐positive cells. (*p < 0.05; **p < 0.01; ***p < 0.001).

The results of HE staining (**Figure** **7**A) show no obvious pathological changes in the sham group. The thickness of the cartilage layer was measured quantitatively by HE. Compared with the OA group, the HA/Wnt16@MSN group shows remarkable improvement in cartilage thickness at 4 and 6 weeks.

The chondro‐protein polysaccharide is stained red and the collagen is stained green in the OA group (Figure 7B). The lighter color of the stained area in the OA group suggests that the entire cartilage matrix in the OA group gradually deteriorates with the progression of the disease, and the distribution of chondrocytes is not uniform. Compared with the other groups, at 2, 4, and 6 weeks, the HA/Wnt16@MSN group shows a wider staining area and brighter and more uniform staining.

Aggrecan immunohistochemical (IHC) staining (Figure 7C) shows a small number of brownish‐yellow granules in the cell matrix of the OA group, suggesting little aggrecan secretion. For the HA/Wnt16@MSN group, the cell matrix shows brownish–yellow granules deposited with more uniform staining, and positive granules appeared in the cell pulp and intercellular stroma, suggesting that the cells are in different periods of aggrecan synthesis and secretion. Aggrecan immunostaining for the HA/Wnt16@MSN group is strongly positive in the ECM as well as the cytoplasm, which indicates an increase in proteoglycan accumulation. The results for Col2 (Figure S15, Supporting Information) show similar trends to those for aggrecan.

IHC outcomes show that Wnt16 (Figure S16, Supporting Information) is barely expressed in healthy articular cartilage. However, more abundant Wnt16 staining in the superficial and calcified cartilage layer of the condyle is observed after 12 weeks than after 4 weeks of treatment with HA/Wnt16@MSN, implying that Wnt16 may be more significant in TMJOA's later phases. Interestingly, in contrast to our results, Liu et al.^[^ ^14^ ^]^ showed that Wnt16 is elevated 4 weeks after OA start and decreases 12 weeks later.

**Figure 8:**
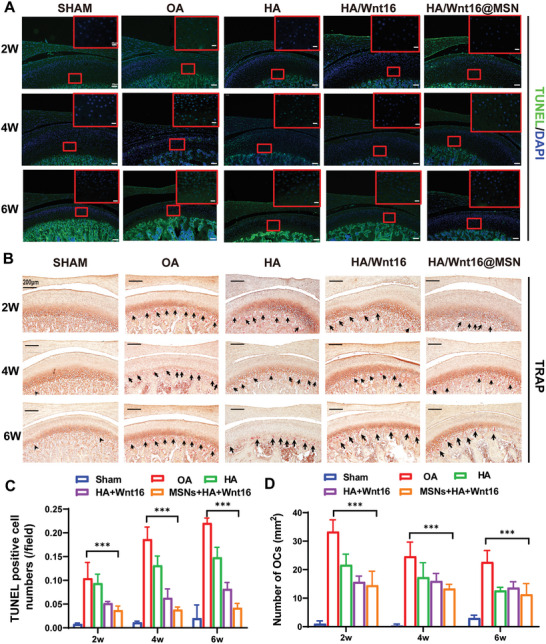
Histological analysis therapeutic effect of HA/Wnt16@MSN. Representative images A) and quantitative analysis C) of TUNEL staining of chondrocytes in various treatment groups. Representative images B) and quantitative analysis D) of TRAP staining of condyle in various treatment groups. (*n* = 6, *p < 0.05; **p < 0.01; ***p < 0.001).

As the components of the composite are added, the number of apoptotic cells gradually decreases, and the full HA/Wnt16@MSN composite has the most significant inhibitory effect. TUNEL staining (**Figure** **8**A) and its quantitative analysis (Figure 8C) likewise indicated that apoptosis of chondrocytes is alleviated to varying degrees after the addition of HA, HA/Wnt16, and HA/Wnt16@MSN. Moreover, changes in condylar subchondral bone were assessed by TRAP staining, the number of OCs (Figure 8B) was significantly reduced after HA/Wnt16@MSN injection. Quantitative counts of the number of apoptotic chondrocytes and OCs are shown in Figure 8C,D.

### 2.9 Effects of HA/Wnt16@MSN on Wnt/β‐Catenin Signaling Pathways

**Figure 9:**
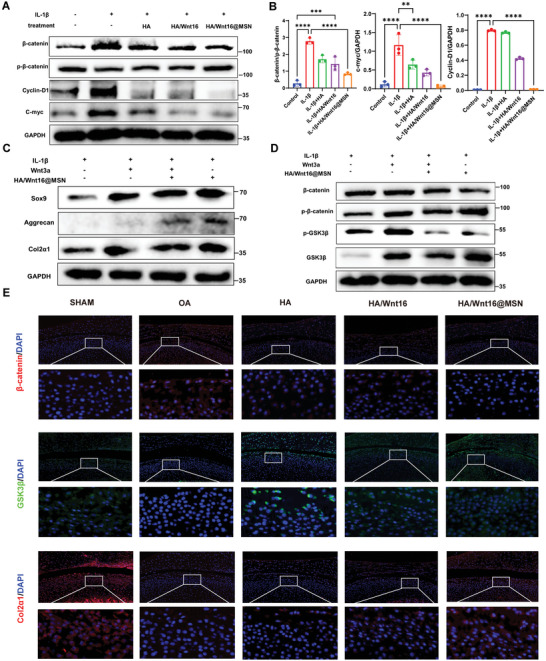
Cartilage repair and anti‐inflammatory mechanism of HA/Wnt16@MSN. A) Western blotting showing the expression of inflammatory‐related proteins and the activation of Wnt/βcatenin pathways with the indicated treatment. B) Quantitative analysis of the protein bands in (A). C,D) Western blotting showing the expression of proteins that related to cartilage matrix and Wnt/β‐catenin signaling with the indicated treatment. E) Representative images of immunostaining of β‐catenin, GSK‐3β, and COL2 6 weeks after indicated injection. (*n* = 3, *p < 0.05; **p < 0.01; ***p < 0.001).

IL‐1β activates Wnt/β‐catenin (**Figure** **9**A,B) signaling pathways in chondrocytes, but treatment with HA/Wnt16@MSN suppresses β‐catenin to reduce Wnt/β‐catenin activation. (Figure 9C) Upon addition of the Wnt/β‐catenin pathway activator Wnt3a, the expressions of genes related to cartilage matrix formation (Sox9, Col2, aggrecan) are downregulated (Figure 9D), indicating HA/Wnt16@MSN alleviates chondrocytes inflammation by inhibiting the Wnt/β‐catenin pathway. To further confirm that the anti‐osteoarthritic effect exerted by HA/Wnt16@MSN is due to its ability to attenuate overactivation of the Wnt pathway, we performed immunofluorescence staining of Col2, GSK3β, and β‐catenin for different groups. The results show that β‐catenin positive chondrocytes are significantly reduced after the injection of HA/Wnt16@MSN, with a significant increase of Col2, GSK3β‐positive cells, which indicated that HA/Wnt16@MSN efficiently regulated Wnt/β‐catenin signaling and inhibited inflammation (Figure 9E).

## 3 Conclusion

In summary, we have developed a novel, multi‐functional, controllable two‐stage delivery system for the treatment of TMJOA in which Wnt16 is loaded into MSNs which, in turn, are loaded into an HA‐based hydrogel, providing a spatially controlled slow release of Wnt16. We confirmed the outstanding biocompatibility of MSNs and their capacity to enhance chondrocyte anabolic activity. Furthermore, Wnt16 demonstrates protective effects against IL‐1β‐induced inflammatory responses in chondrocytes by promoting cartilage anabolism and mitigating the IL‐1β‐induced suppression of cartilage catabolism. HA/Wnt16@MSN modulates chondrocyte activation by inhibiting the Wnt/β‐catenin pathway in chondrocytes under inflammatory conditions. Furthermore, animal experiments indicated that HA/Wnt16@MSN effectively reduces osteophytes formation while protecting articular cartilage and subchondral bone structure.

However, it is important to note that our study has some shortcomings, one being that the localization of Wnt16 in the articular cartilage layer is not in the same location as previously reported.^[^ ^15^ ^]^ This may be due to the fact that the method in the previous study was to inject Wnt16 directly into the joint cavity, while the present study used a double‐layer structure to slow‐release Wnt16, which could allow it to not only positively regulate cartilage, but also inhibit the osteoblastic changes occurring in the subchondral bone during the process of TMJOA, so as to have a synergistic effect. Secondly, the animal experiments in this study were only carried out up to the sixth week, so the long‐term and stable efficacy of HA/Wnt16@MSN requires a longer course of treatment to be established in the future. Furthermore, our disease model was only explored for HA/Wnt16@MSN alleviation of TMJOA in rats, and further animal experiments (e.g., large animals such as goats) are required to validate its effect and to extend it to the clinic.

In conclusion, our study suggests that HA/Wnt16@MSN achieves sustained controlled drug release and efficient chondrocyte anabolic and anti‐inflammatory activity. Consequently, HA/Wnt16@MSN has excellent application potential for the effective treatment of TMJOA.

## 4 Experimental Section

### Materials

Recombinant Human Wnt‐16b Protein was purchased from R&D Systems (Minneapolis, MN, USA). Hexadecyltrimethylammonium bromide (CTAB, 98%) and chlorhexidine were purchased from Sigma–Aldrich (Shanghai, China); tetraethyloxysilane (TEOS) and sodium hydroxide (NaOH) were obtained from Sinopharm Chemical Reagent Limited Corporation and used as received. Hyaluronic acid (HA; MW = 100–200 kDa) was purchased from Bloomage Biotechnology Corporation Limited.

Trypsin‐EDTA (0.25%), fetal bovine serum (FBS), Dulbecco's modified eagle medium/nutrient mixture F‐12 (DMEM‐F12), and phosphate buffered saline (PBS, pH = 7.4) were all purchased from GIBCO BRL (Grand Island, NY, USA). Recombinant mouse RANKL and macrophage colony‐stimulating factor (M‐CSF) were purchased from R&D (Minneapolis, MN, USA).

### Synthesis of MSNs

The MSNs were prepared and synthesized according to the literature with subtle adjustments. Briefly, 0.1 g of CTAB was dissolved in 480 mL of water, and 3.5 mL of 0.2 m NaOH was added. The resulting solution was heated to 85 °C and stirred vigorously for 1 h. Subsequently, 0.5 mL of TEOS was added dropwise, and the mixed solution was stirred for an additional 1 h. The MSNs were acquired through a centrifugation process, subjected to multiple ethanol washes, and then centrifuged to remove residual ethanol. To eliminate the surfactant CTAB, the MSNs were refluxed in HCl/methanol for 24 h. Finally, the MSNs obtained by centrifugation were dried overnight at 60 °C under vacuum to expel solvent residues from the mesopores, resulting in the production of mesoporous materials.

### Physicochemical Characterization

All the samples were vacuum freeze–dried before material characterization. The pore structure and particle size of the MSNs were analyzed by transmission electron microscopy (TEM; FEI Company, USA), and the morphologies of the materials were observed by scanning electron microscopy (SEM; FEI Company, USA). The specific surface area and pore size distribution of MSN nanomaterials were determined by N_2_ adsorption–desorption experiments. BET measurement and BJH analysis were used to determine the surface areas and the pore size distributions of different particles.

### Synthesis of HA/Wnt16@MSN

MSNs of 60 mg were dispersed in 4 mL PBS, followed by the addition of 2200 µL PEI solution (75 mg mL^−1^). The mixtures were stirred for 30 min at room temperature. The product was then centrifuged (10 000 rpm, 15 min) and washed three times with ultrapure water to obtain PEI‐MSN. Then 30 mg HA, 80 mg NHS, and 60 mg EDC were dissolved in 20 mL distilled water and stirred for 1 h to activate the carboxyl groups in the hyaluronic acid. PEI‐MSN was then added to the mixture and stirred for a further 24 h at room temperature. Finally, HA‐MSN was obtained after centrifugation, washing, and overnight lyophilization. Then, HA‐MSNs (100 mg) were immersed in 10 mL PBS containing 100 ng mL^−1^ Wnt16. A small vacuum pump was used to evacuate air trapped within the pores of the MSNs, assisting the incorporation of Wnt16. Upon visual confirmation of the removal of air bubbles from the MSNs, they were dried in air at room temperature, and prepared for drug‐release testing.

### Drug‐Release Analysis

A quantity of MSNs loaded with Wnt16 was introduced into a centrifuge tube containing 8 mL of PBS with a pH of 7.4. Subsequently, the centrifuge tube was positioned in a constant temperature oscillating incubator at 37 °C with a rotation speed of 120 rpm. At specific time intervals (0, 24, 48, 96, 120, 144, 168, 192, 216, and 240 h), 1 mL of the sample was withdrawn, and the sample was replenished with an equivalent volume of PBS maintained at the same temperature and pH. The withdrawn samples underwent centrifugation at 13 000 rpm and 25 °C for 10 min, following which the supernatant was collected for analysis. Each experiment was replicated three times to ensure precision. Upon completion of the sampling process, the concentration of the supernatant was quantified using an enhanced BCA protein assay kit, and the OD value of each solution was determined by scanning the characteristic absorption wavelength using an enzyme standardizer. A standard curve was drawn according to the OD value. Finally, the cumulative release curve was meticulously constructed using dedicated plotting software. The experiment of slow release of Wnt16 by HA alone required the configuration of a mixture of 10 mg mL^−1^ HA containing 2 mg mL^−1^ Wnt16, and 1 mL of the HA‐Wnt16 mixture was taken into a 2 mL dialysis bag (1000 kDa) and then placed into a 10 mL PBS slow‐release system for the release and assayed. The release of Wnt16 by HA in a 10 mL PBS slow‐release system was measured in a 10 mL PBS system.

### Cell Culture

Human chondrocytes (SW1353 cells) were procured from the American Type Culture Collection (ATCC). These cells were carefully cultured in DMEM‐F12 supplemented with 10% FBS, 100 µg mL^−1^ streptomycin, and 100 U mL^−1^ penicillin. The cultivation process occurred in a humidified incubator set at 5% CO_2_ and 37 °C. Following incubation, cell harvesting was achieved through gentle scraping followed by centrifugation. The resultant cell pellet was resuspended in a fresh medium, and the cell count was determined using the standard trypan blue cell counting technique.

BMMs were harvested from the fresh femurs of six‐week‐old C57/BL6 mice using the following: Initially, the euthanized mice were immersed in 75% ethanol for 5 min for sterilization. Next, the surrounding soft tissues of both lower extremities were cleaned, and the femur and tibia were separated, followed by double washing with DMEM medium. Subsequently, the bone ends were trimmed with surgical scissors on an ultra‐clean bench, and the bone marrow cavity was flushed with DMEM and filtered through a 70 µm cell strainer. After the addition of red blood cell lysate, the mixture underwent centrifugation at 1000 rpm for 5 min to eliminate the supernatant. The resulting pellet was resuspended and cultured in DMEM medium supplemented with 10% FBS, 50 ng mL^−1^ M‐CSF (462‐TEC‐010/CF, R&D Systems, Minneapolis, MN, USA), and 100 µ mL^−1^ Penicillin–Streptomycin–Amphotericin B. The medium was changed every other day prior to seeding and drug intervention.

### Screening for Optimal Concentrations of MSNs and Wnt16

Cells were harvested when the population reached 80% proliferation. Cell cytotoxicity was assessed through CCK‐8 assays. Briefly, SW1353 cells were enumerated and seeded at a density of 10^4^ cells per well in 96‐well plates, each containing 100 µL of complete medium. Subsequently, the cells were left to adhere overnight in a 37 °C incubator with 5% CO_2_. Following adherence, a PBS wash was performed, and fresh complete medium supplemented with varying concentrations of MSNs (0, 1.56, 3.125, 6.25, 12.5, 25, 50, 100, 150, 200 µg mL^−1^) and Wnt16 (0, 25, 50, 100, 200, 400 ng mL^−1^) were introduced to the wells. The cells were then incubated for an additional 48 h. After incubation, the medium was aspirated, and the cells were rinsed with PBS. Subsequently, 100 µL of FBS‐free medium with 10% CCK‐8 was added to each well. After 2 h, the medium underwent a color change to brown or yellow. The cell cytotoxicity of MSNs and Wnt16 was assessed by measuring absorbance at 450 nm using a microplate reader (Thermo Scientific, Waltham, MA, USA). Additionally, live/dead staining was employed to evaluate cell viability.

### Quantitative PCR Analysis

To evaluate the expression of inflammation‐related genes and genes related to cartilage synthesis and degradation, SW1353 cells were seeded in six‐well plates at a density of 4 × 10^5^ cells/well and then cultured with IL‐1β under various treatment conditions for 24 h. TRIzol (TaKaRa, Tokyo, Japan) was used for total RNA extraction according to the manufacturer's instructions. RT‐qPCR assay was performed on an ABI 7500 Sequencing Detection System (Yeasen, Shanghai, China) using the SYBR® Premix Ex Taq II according to the manufacturer's instructions after reverse transcription from RNA templates. The sequences of related primers used are listed in Table S1 (Supporting Information).

### Western Blot

SW1353 chondrocytes were seeded at a density of 4 × 10^5^ cells per well in six‐well plates. After coculture with various treatment conditions for 48 h, total protein extraction was performed using whole cell lysis buffer supplemented with a protease inhibitor cocktail (P8340; Sigma–Aldrich). The proteins were separated by 10% SDS‐PAGE after being dissolved in loading buffer, and subsequently transferred to 0.22 µm polyvinylidene fluoride (PVDF) membranes. After blocking, incubation with primary antibodies (GAPDH, 1:10000; Col1a1, 1:1000; Sox9, 1:1000; Col2a1, 1:1000; Aggrecan, 1:1000; MMP13, 1:1000; p‐P65, 1:1000; p65, 1:1000; IκB, 1:1000; β‐catenin, 1:1000; GSK‐3β, 1:1000) was performed overnight at 4 °C. Subsequently, incubation with HRP‐conjugated secondary antibodies (1:10000, AS014, Abclonal, China) for 1 h at room temperature was carried out, and the blots were visualized by Image Lab V3.0 image scanning (Bio‐Rad Laboratories, Hercules, Calif).

### Flow Cytometry

The apoptotic effect of SW1353 chondrocytes was explored with Annexin V‐FITC/PI kits (Abcam, ab14085) and a FACS LSRFortessa flow cytometer (BD Biosciences, Franklin Lakes, NJ, USA).

Quantitative analysis of fluorescence intensity was carried out through flow cytometry. SW1353 cells were counted and seeded in six‐well plates as previously, After adhesion, different treatment reagents were added separately as follows: 1) negative control; 2) negative control +IL‐1β; 3) negative control +IL‐1β+HA; 4) negative control +IL‐1β+Wnt16; 5) negative control +IL‐1β+MSN; 6) negative control +IL‐1β+Wnt16+MSN; 7) negative control +IL‐1β+HA+Wnt16+MSN (concentrations of each component as previously described). After 48 h, the cells were washed twice with PBS and incubated for 15 min at room temperature and in the dark with PI before being stained with Annexin V‐FITC for 15 min at 4 °C for flow cytometry analysis. The apoptotic cells were detected, and the apoptosis percentage under different treatments was calculated.

### TRAP Staining Assay and Bone Resorption Assay

BMMs were seeded at 1 × 10^4^ cells per well into 96‐well plates, followed by culture with M‐CSF (30 ng mL^−1^) and RANKL (100 ng mL^−1^) in the presence of either the composite or its constituents (HA, HA/Wnt16, HA/Wnt16@MSN). The culture medium underwent renewal every 2 days until the observation of OC formation after 5 days. For the TRAP staining assay, following fixation for 20 min with 4% paraformaldehyde (PFA), the cells were subjected to staining with TRAP solution at 37 °C for 1 h. OCs were identified as TRAP‐positive cells containing more than three nuclei. Image capturing was performed using an optical microscope (Olympus, Tokyo, Japan), and the quantification of OCs number and area was conducted using ImageJ software (NIH, Bethesda, MD, USA). For bone resorption assay, following an additional day of culture, mature OCs were eliminated using 5% sodium hypochlorite. Subsequently, optical microscopy was employed to image the resorption pits, and the bone resorption area was quantified using Image J software.

### Establishment of the TMJOA Model

The animal protocol was approved by the Animal Care and Experiment Committee of Ninth People's Hospital Affiliated to Shanghai Jiao Tong University School of Medicine (SH9H‐2024‐A12‐1). The TMJOA model based upon previous studies was established for the evaluation of various treatment groups in vivo.^[^ ^34^ ^]^ Briefly, unilateral anterior crossbite (UAC) stimulation was applied to 45 six‐week‐old male SD rats with a pair of metal prosthetics. After the application of the UAC device, the inspection was carried out to avoid shedding. No device became detached during the experimental period. Then, the 45 rats were randomly divided into five groups (each group contained three rats, *n* = 6): 1) Sham group; 2) UAC group; 3) HA group (10 mg mL^−1^); 4) HA/Wnt16 group (the concentration of Wnt16 was 100 ng mL^−1^, HA as above); 5) HA/Wnt16@Msn treated group (the concentration of MSNs was 100 µg mL^−1^; Wnt16 and HA as above). Bilateral TMJ cavity injections were started one week after UAC application in all groups except the UAC group. The sham‐operated group was injected with saline (50 µL side^−1^) and the remaining groups were administered weekly. Three rats from each group were sacrificed on week 2, week 4, and week 6, and the joint structures were dissected, photographed, and fixed in 4% PFA for 48 h for further analysis.

### Micro‐CT

A high‐resolution micro‐CT apparatus (µCT‐100, SCANCO Medical AG, Switzerland) with a resolution of 10 µm was utilized to observe the condylar morphology and morphometric changes of bone histology in the rats. The X‐ray energy was set at 80 kV and 200 µA. The ROI (Region of Interest, ROI) selection method was carried out in accordance with the literature.^[^ ^35^ ^]^

### Histological and Immunohistochemical Analysis

After micro‐CT scanning, all samples were decalcified in 10% EDTA (pH = 7.4) for two months. After embedding, histological sections were prepared for H&E staining, safranin O‐fast green (S&F) staining, and TRAP staining. IHC staining was performed with antibodies against aggrecan (1:100; PA1‐036), Col‐X (1:100; AF0639), Wnt16 (1:100; PAB26056) and Col2 (1:200, ab34712). Following, percentage of positive cells in the cartilage layer was quantified by Image J.

### Immunofluorescence Analysis

After 0.05% trypsin retrieval and blocking, the sections were incubated with primary immunofluorescence antibodies consistent with 4.9 experiment at 4 °C overnight. The next day, the slides were incubated with HRP/Alexa Fluor 594‐conjugated secondary antibodies (a24421, a24211, Abbkine, USA) for 1 h at 37 °C. DAPI was applied for the nucleus staining.

### Statistical Analysis

All data are displayed as mean ± SD. GraphPad Prism 9.0 software (GraphPad, San Diego, CA, USA) was used for data analysis. Statistical significance was assessed using a one‐way analysis of variance (ANOVA) followed by Tukey test (*p < 0.05; **p < 0.01; ***p < 0.001; ****p < 0.0001).

## Conflict of Interest

The authors declare no conflict of interest.

## Author Contributions

Y.Z., L.Y.C., and M.Y contributed equally to this work. ZG.M., XS.W., and C.Y. contributed to experiment design, execution, and interpretation; Y.Z., M.Y. X.Z.C., X.R.X., M.H.L., and L.Y.C. performed the experiments; Z.G.M, Y.Z., M.Y. and L.Y.C. contributed to study design and interpretation; Z.G.M. and X.S.W. oversaw the collection of results and data interpretation, and drafted the manuscript. C.Y. designed the experiments, oversaw the collection of results and data interpretation, and drafted the manuscript. All authors have seen and approved the final version.

## Supporting information

Supporting Information

## Data Availability

The data that support the findings of this study are available from the corresponding author upon reasonable request.
