# Supplementary material for: Microgel Encapsulated Mesoporous Silica Nanoparticles for Releasing Wnt16 to Synergistically Treat Temporomandibular Joint Osteoarthritis
Source: Adv Sci (Weinh). 2024 Sep 9;11(41):2404396. doi: 10.1002/advs.202404396 (PMC11538678; doi:10.1002/advs.202404396)
Supplement: Supplementary file 1 — Supporting Information [file ADVS-11-2404396-s001.docx]

**Supporting Information**

This supporting information includes Fig. S1-18 and Table S1.


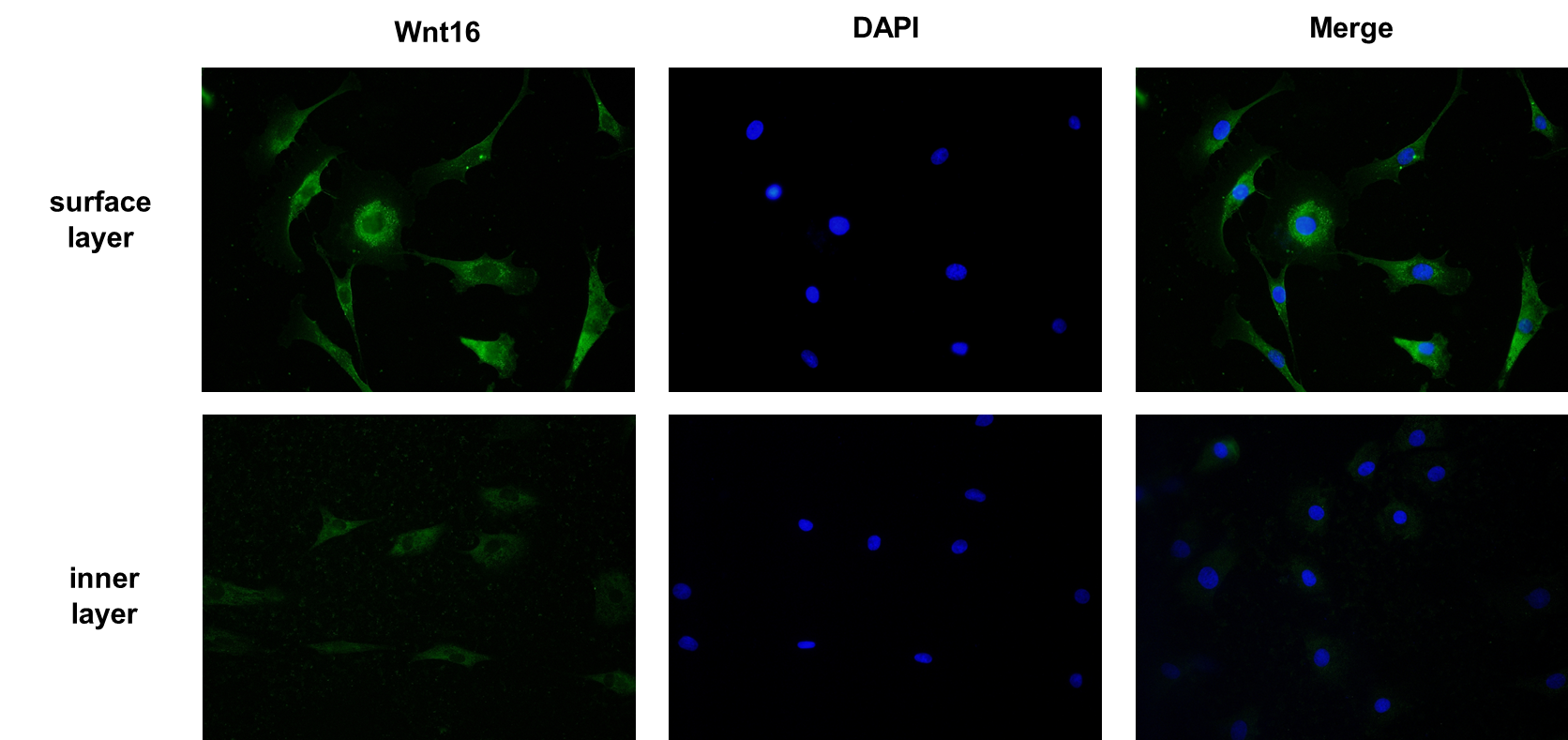


Figure S1. Representative IF images showing the expression and location of Wnt16 in the cartilage cells of the rat TMJ condyle.


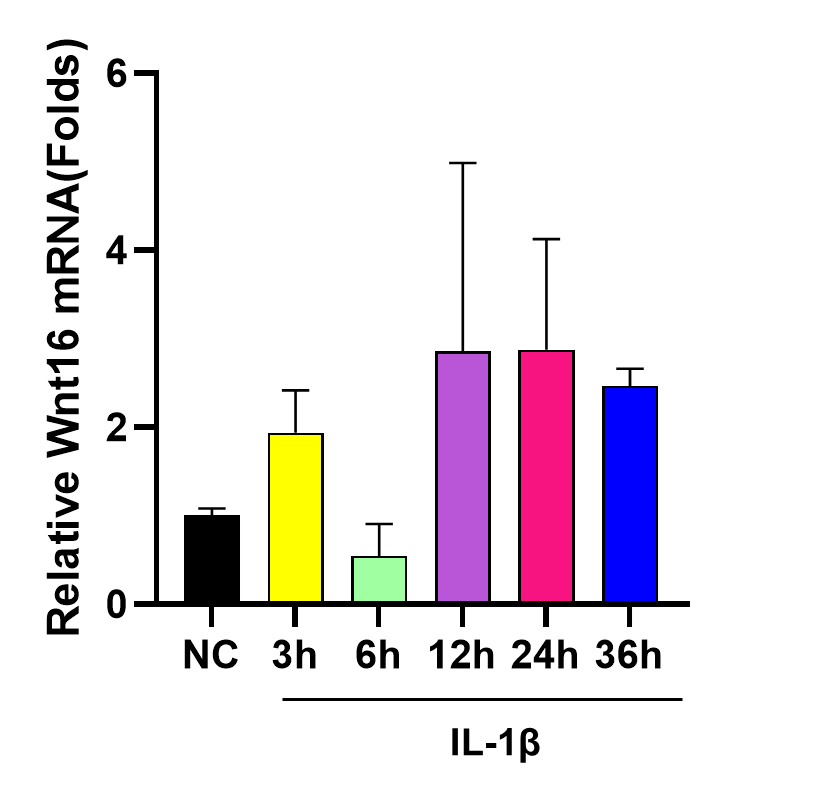


Figure S2. Expression levels of Wnt16 mRNA in SW1353 chondrocytes after IL-1β (10 μg/mL) activation for 3, 6, 12, 24, and 36 h.


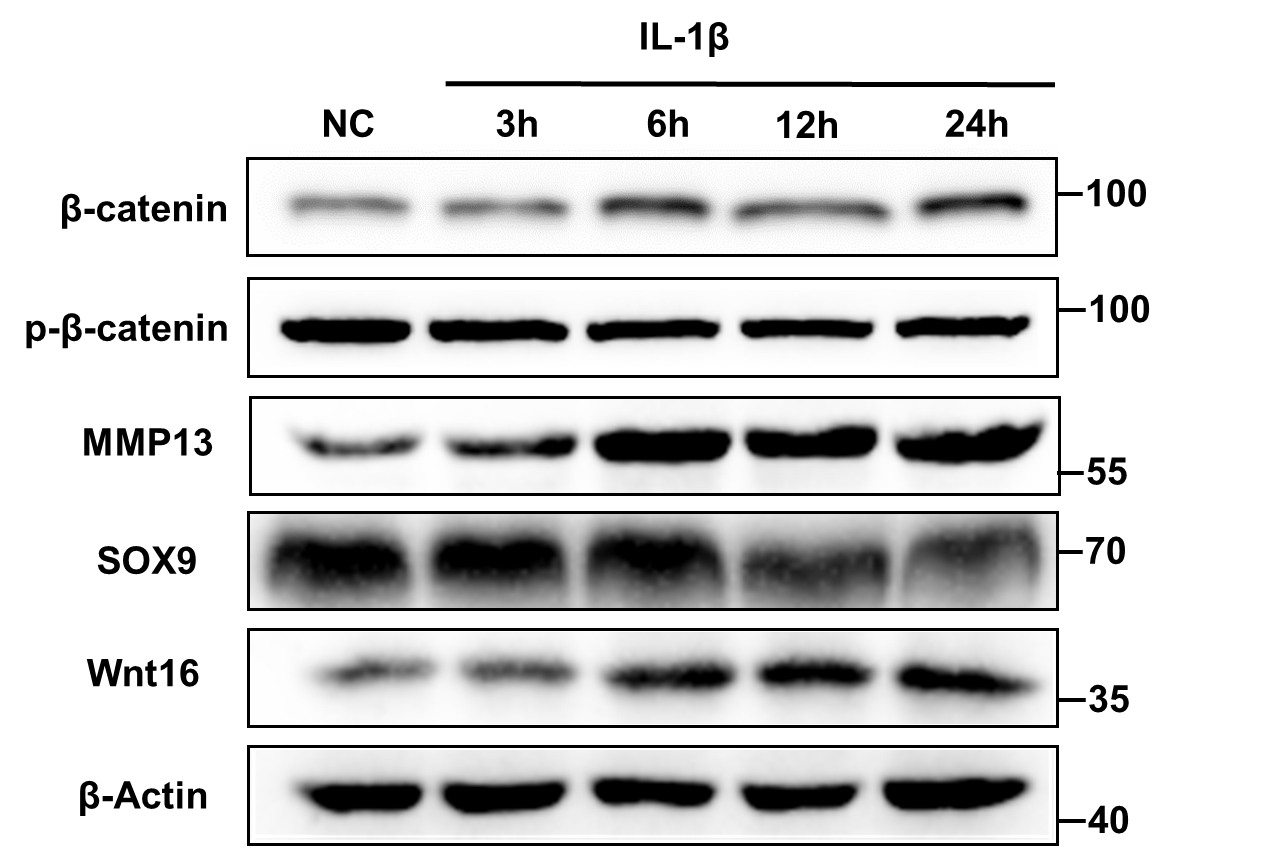


Figure S3. Expression levels of Wnt16, cartilage inflammatory, and cartilage matrix proteins in SW1353 chondrocytes after IL-1β activation for 3, 6, 12, and 24 h.


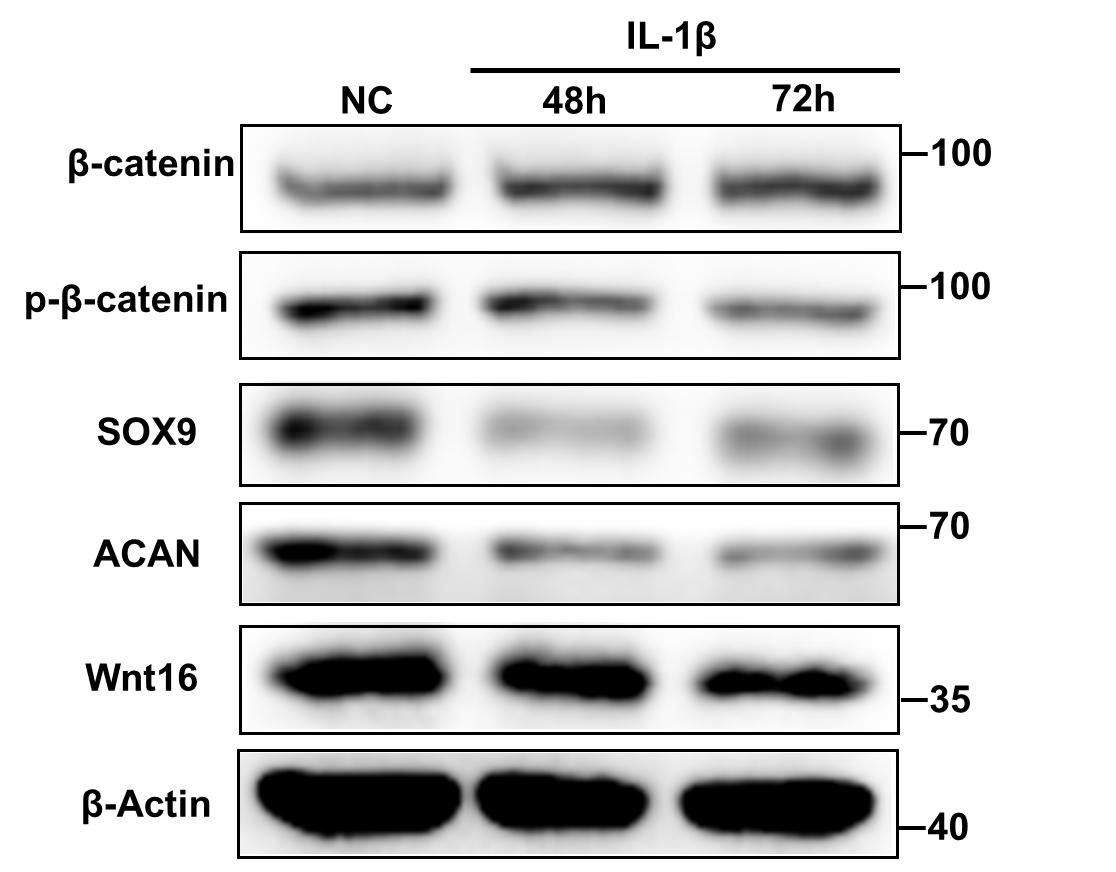


Figure S4. Expression of Wnt16 protein, cartilage inflammatory proteins, cartilage matrix proteins, and Wnt-signaling-pathway-related proteins in SW1353 cells after 48 and 72 h stimulation with 10 μg/mL IL-1β.


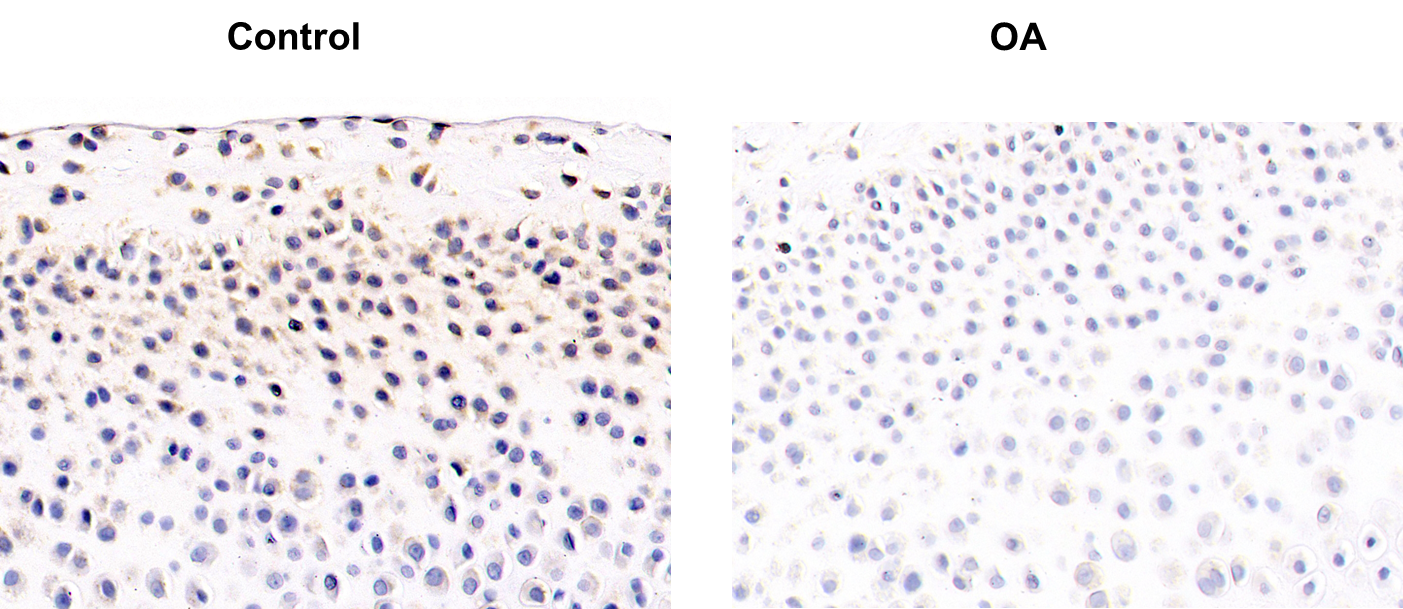


Figure S5. IHC staining of Wnt16 in normal and OA cartilage (scale bar:200 μm).


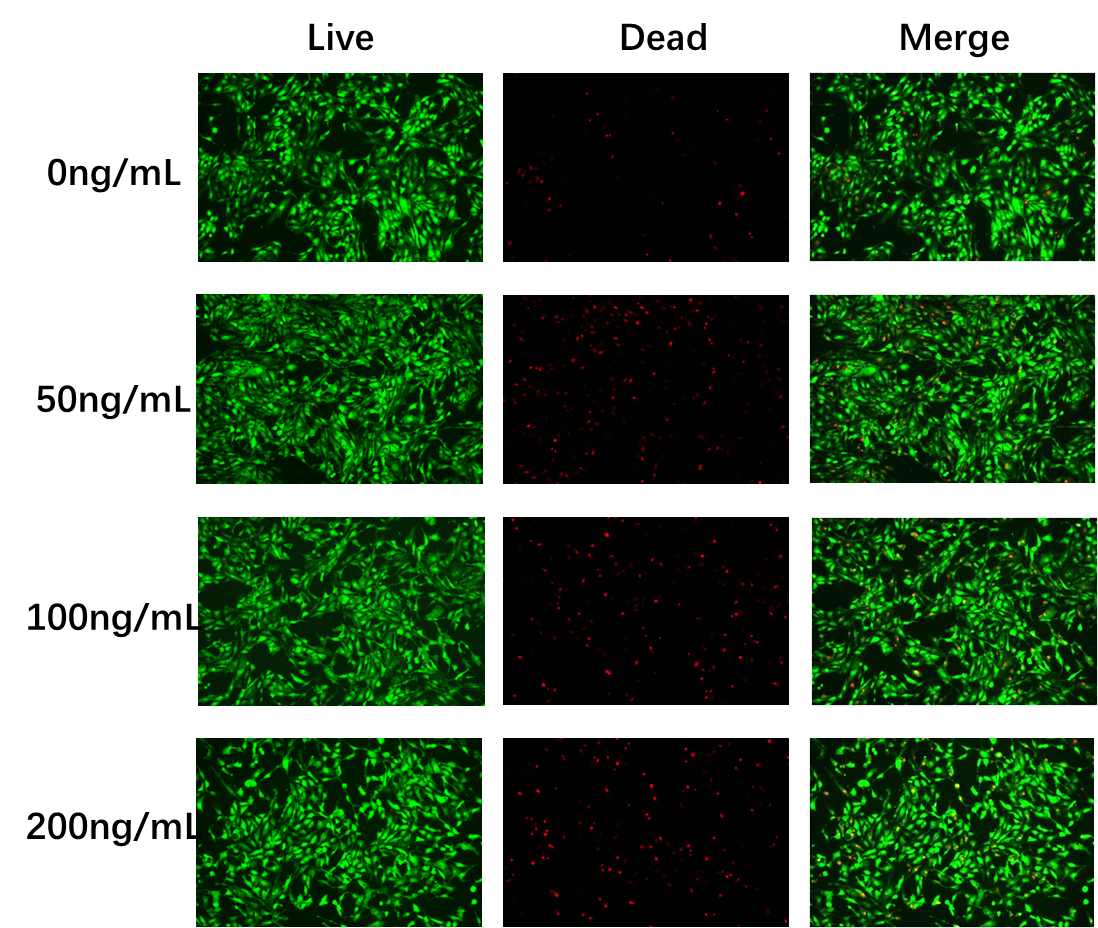


Figure S6. Live/dead staining of SW1353 treated with MSNs at various concentrations for 48 h (scale bar:200 μm).


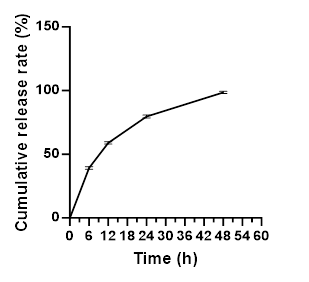


FigS7: Cumulative release curve of Wnt16 encapsulated in HA.


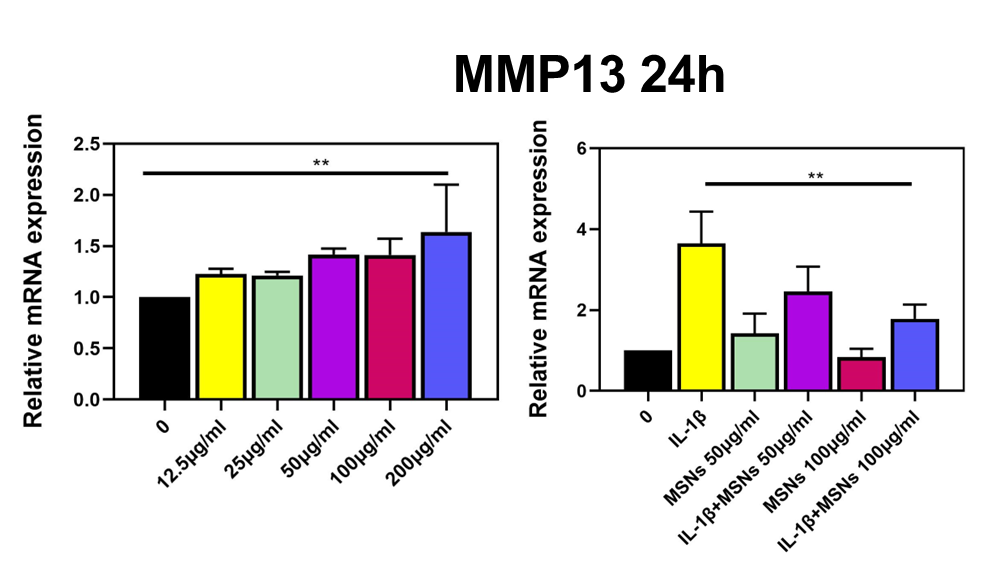


Figure S8. Expression levels of MMP13 mRNA by SW1353 cells treated with MSNs at various concentrations after 24 h stimulation with (or without) 10 μg/mL IL-1β.


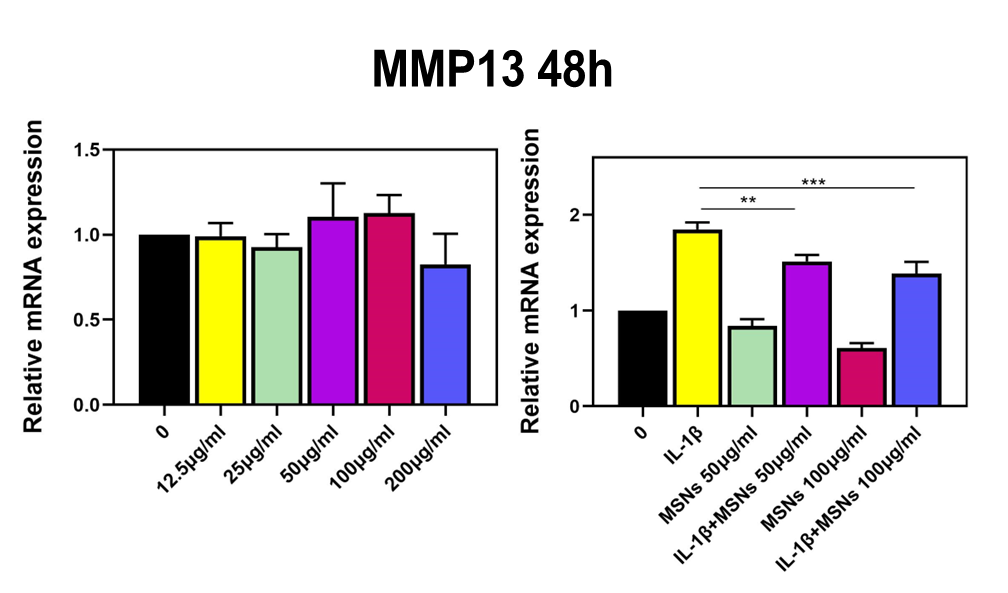


Figure S9. Expression levels of MMP13 mRNA by SW1353 cells treated with MSNs at various concentrations after 48 h stimulation with (or without) 10 μg/mL IL-1β.


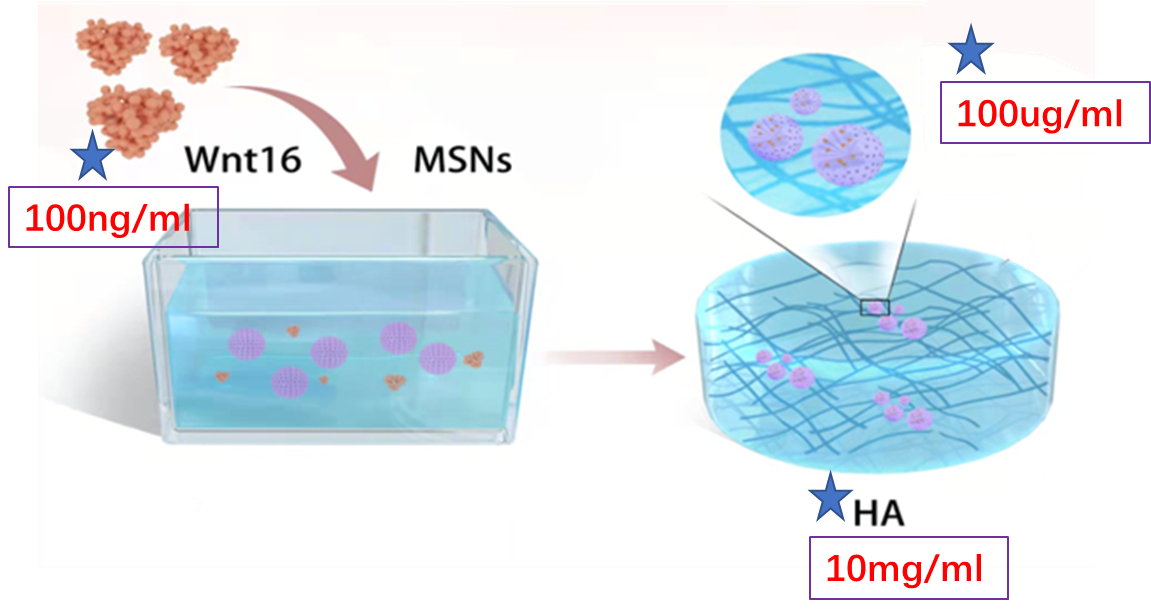


Figure S10. Schematic showing component concentrations and construction of HA/Wnt16@Msn.


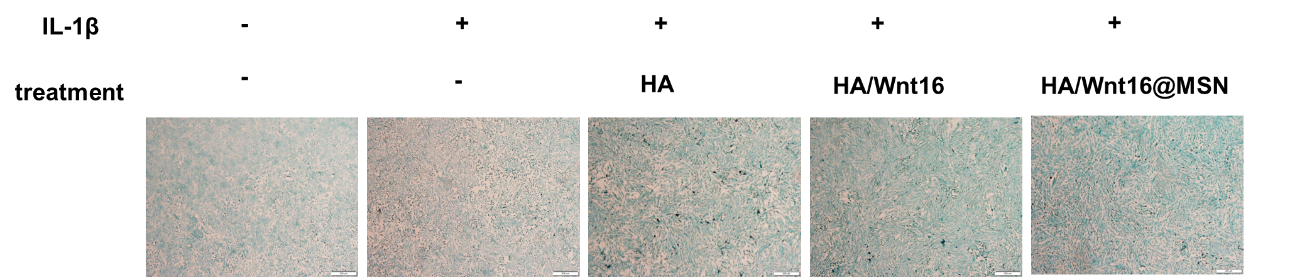


Figure S11. Alcian Blue staining of SW1353 chondrocytes treated with different components of HA/Wnt16@MSN after 48 h (scale bar: 100 μm).


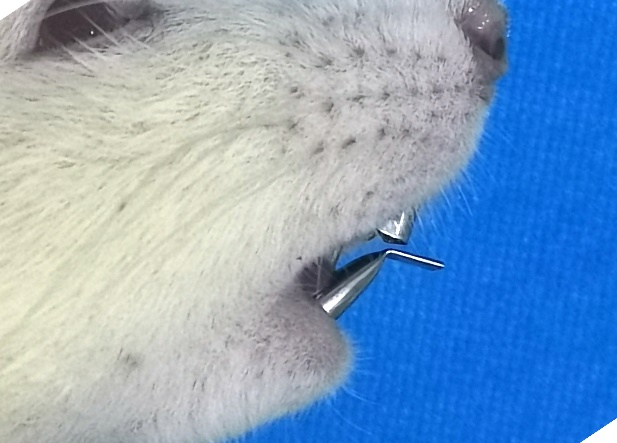

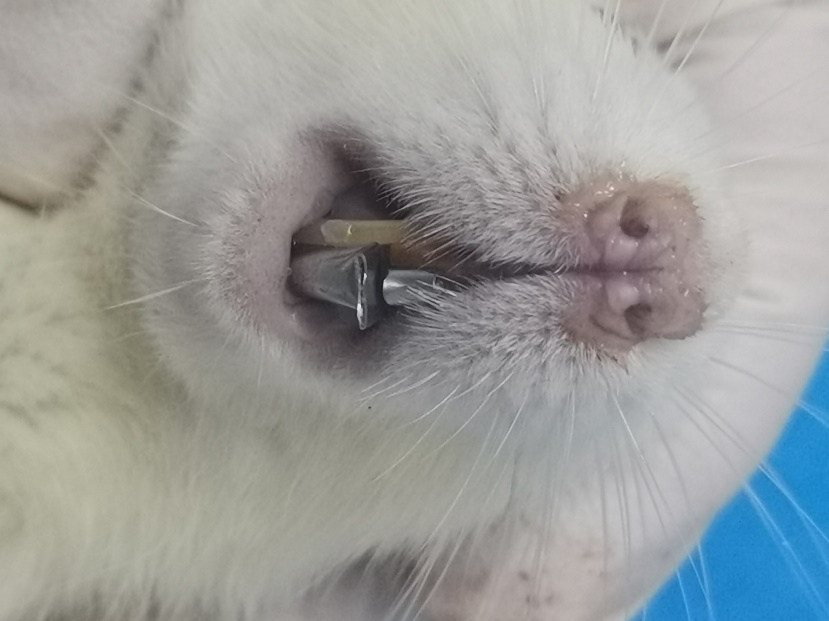


Figure S12. UAC modification used to establish the TMJOA model in SD rats.


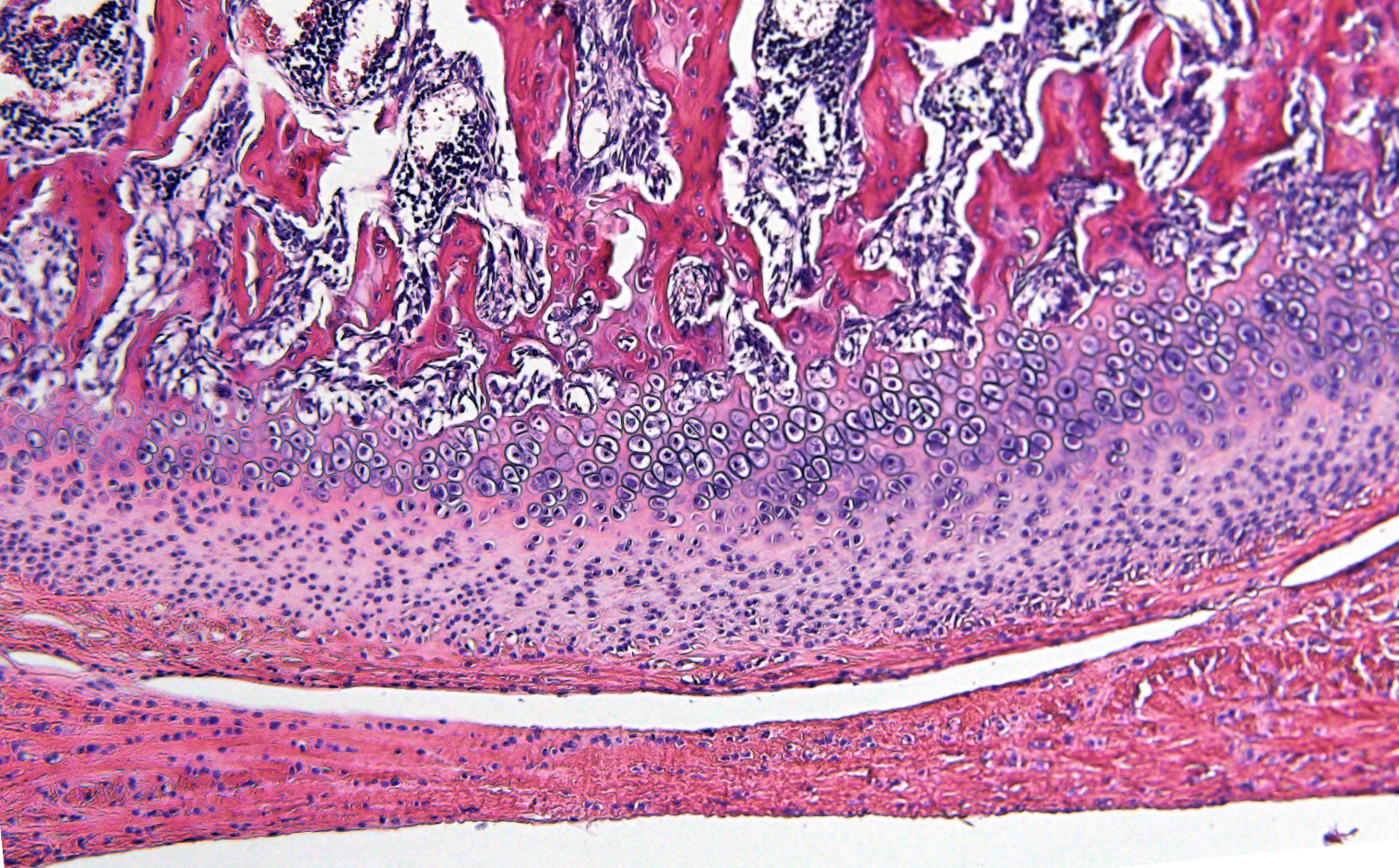


**100µm**

**articular disc**

**condylar**

**cartilage**

Figure S13. Cartilage thickness measurement in sagittal central section of TMJs stained with H&E.


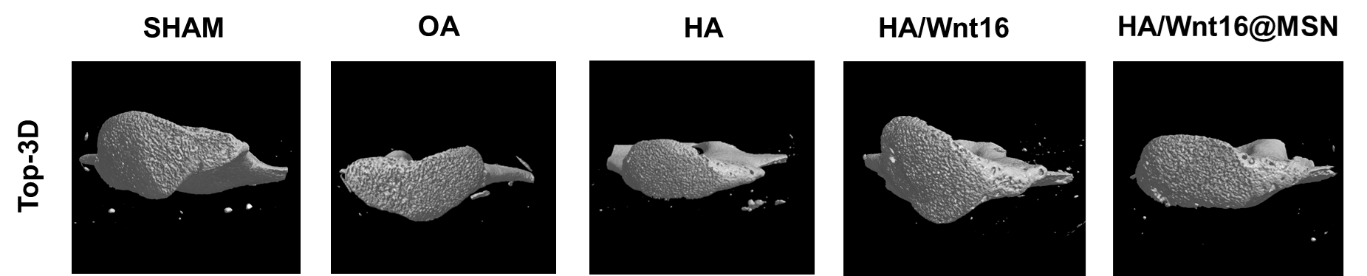


Figure S14. Micro-CT images (top-3D view) of TMJ condyles after injection with HA/Wnt16@Msn and its components.


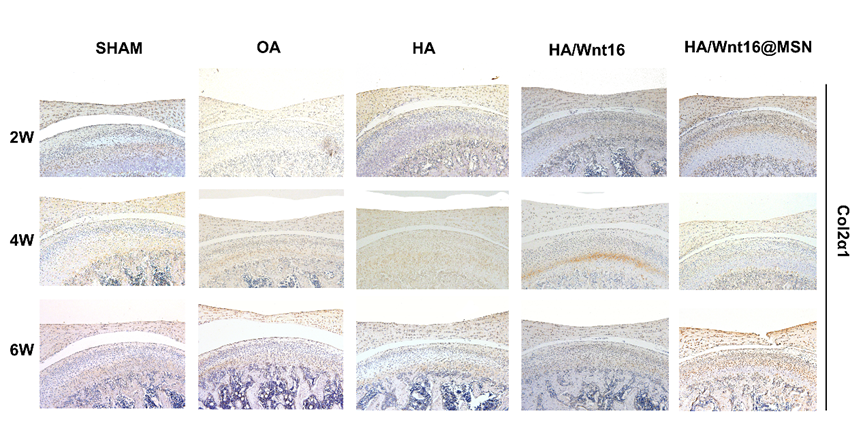


Figure S15. Representative images of IHC staining of Col2 for various treatment groups. (scale bar: 200 μm). n = 6.


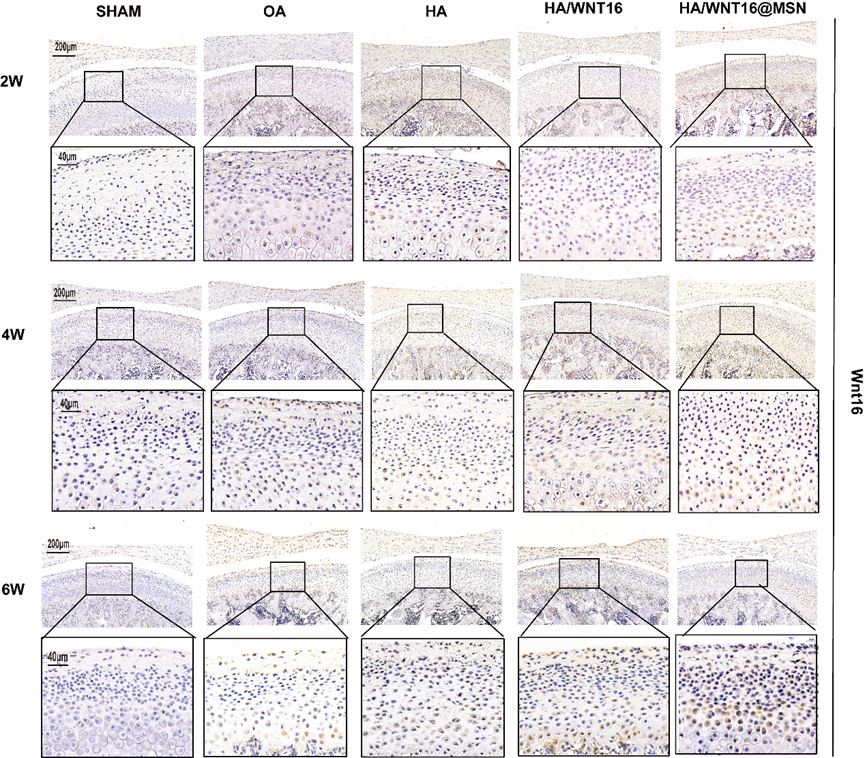


Figure S16. Representative images of IHC staining of Wnt16 for various treatment groups (scale bar: 200 μm). n = 6.


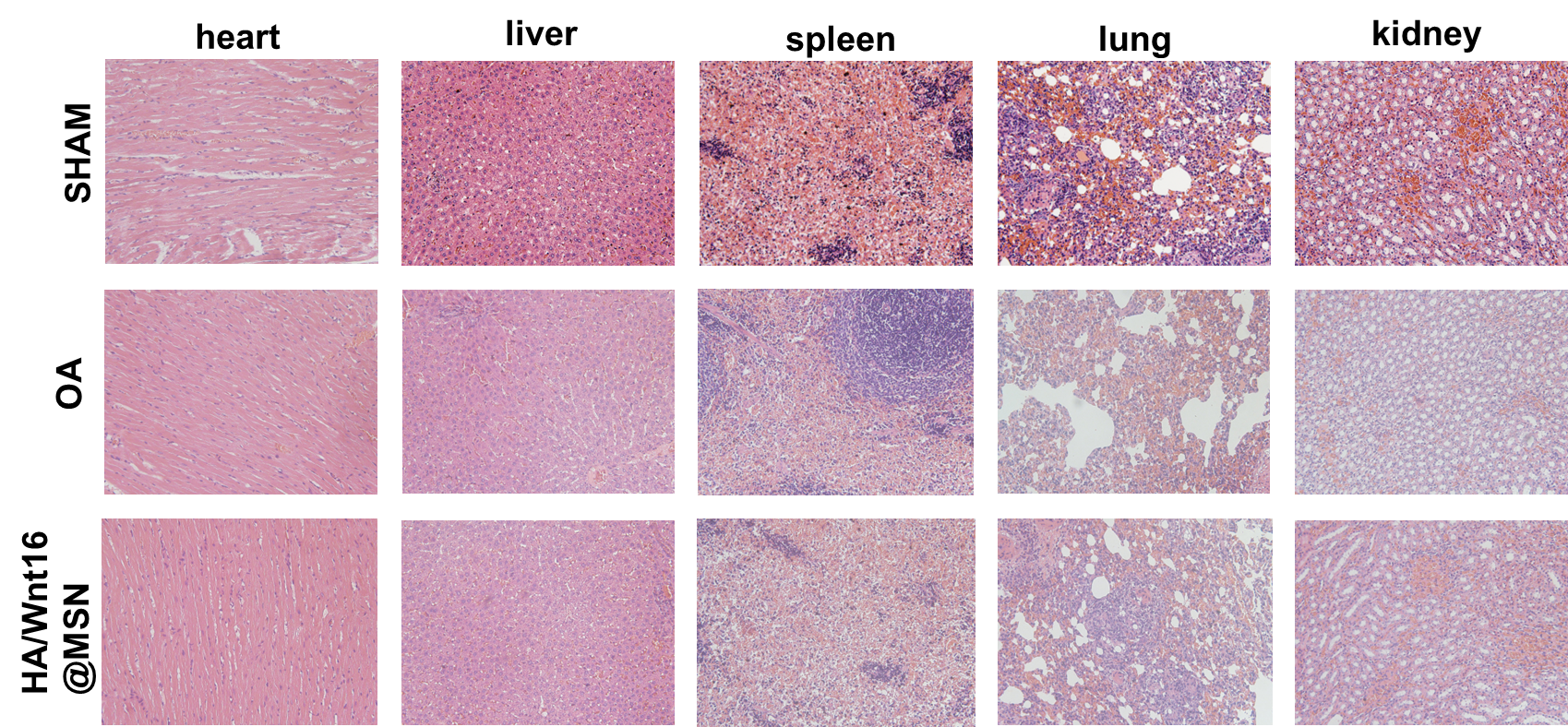


Figure S17. Representative H&E staining images of heart, liver, spleen, lung, and kidney tissues 14 days after model establishment (11 days after hydrogel injection) n = 6. (scale bar: 200 μm).


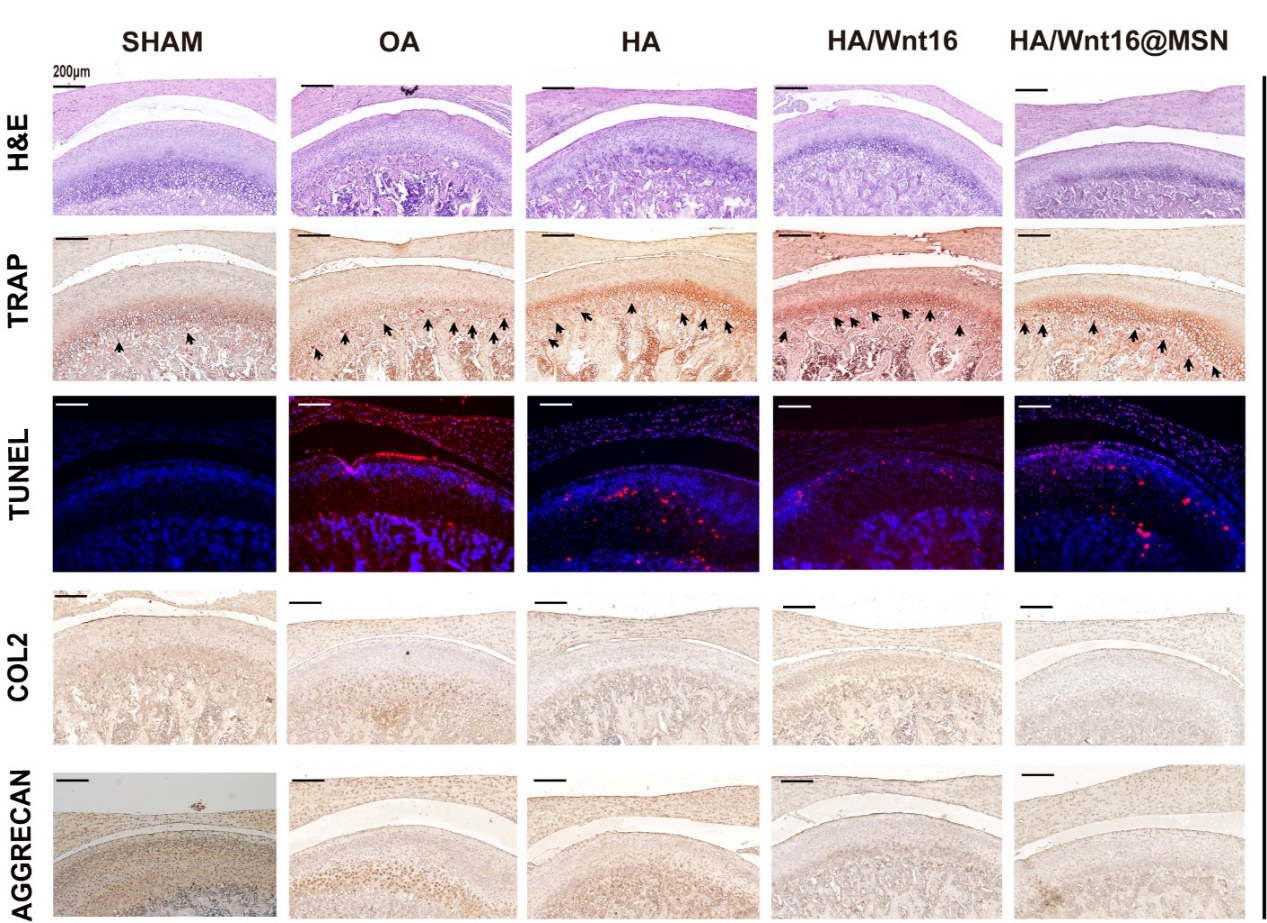


Figure S18 Histological analysis of TMJ prior to Wnt16 injection. Representative images of H&E, TRAP, TUNEL and IHC of aggrecan, col2 of various treatment groups, n = 6. (scale bar: 200 μm).

**Table S1.** Primer sequences used for real-time PCR

| **Name** | **Primer sequence** |
| --- | --- |
| GAPDH | Forward: 5′-CAAGTTCAACGGCACAGTCAAG-3′ |
|  | Reverse: 5′-ACATACTCAGCACCAGCATCAC-3′ |
| Aggrecan | Forward: 5′-TGGCATTGAGGACAGCGAAG-3′ |
|  | Reverse: 5′-TCCAGTGTGTAGCGTGTGGAAATAG-3′ |
| Col2α1 | Forward:5′-GAGGGCAACAGCAGGTTCAC-3′  Reverse: 5′-GCCCTATGTCCACACCAAATTC-3′ |
| MMP13 | Forward: 5ʹ-CTGGCCTGCTGGCTCATGCTT-3ʹ |
|  | Reverse: 5ʹ-CCTCAGAAAGAGCAGCATCGATATG-3ʹ |
| ADAMTS-4 | Forward: 5ʹ-ACACTGAGGACTGCCCAAC-3ʹ |
|  | Reverse: 5ʹ-GGTGAGTTTGCACTGGTCCT-3ʹ |
| ADAMTS-5 | Forward: 5ʹ-GCAGAACATCGACCAACTCTACTC-3ʹ |
|  | Reverse: 5ʹ-CCAGCAATGCCCACCGAAC-3ʹ |
| Sox9 | Forward: 5’- AGGAAGCTCGCGGACCAGTAC-3’ |
|  | Reverse: 5’- GGTGGTCCTTCTTGTGCTGCAC-3’ |
| Col1α1 | Forward: 5’- AGAACAGCGTGGCCT-3’ |
|  | Reverse: 5’- TCCGGTGTGACTCGT-3’ |
| Bcl2 | Forward: 5’- CTGTGCTGCTATCCTGC-3’ |
|  | Reverse: 5’- TGCAGCCACAATACTGT-3’ |
| Bax | Forward: 5’- AGACACTCGCTCAGCTTCTTG-3’ |
|  | Reverse: 5’- CTTTTGCTTCAGGGTTTCATC-3’ |
| Wnt16 | Forward: 5'- AGTATGGCATGTGGTTCAGCA-3' |
|  | Reverse: 5'- GCGGCAGTCTACTGACATCAA-3' |
